# Effectiveness and safety of the combination of sodium–glucose transport protein 2 inhibitors and glucagon-like peptide-1 receptor agonists in patients with type 2 diabetes mellitus: a systematic review and meta-analysis of observational studies

**DOI:** 10.1186/s12933-024-02192-4

**Published:** 2024-03-18

**Authors:** Aftab Ahmad, Hani Sabbour

**Affiliations:** 1 Department of Endocrinology, Imperial College London Diabetes Centre, Abu Dhabi, United Arab Emirates; 2 Department of Endocrinology, Khalifa Medical University, Abu Dhabi, United Arab Emirates; 3 Department of Cardiology, Mediclinic Hospital, Abu Dhabi, United Arab Emirates; 4 Department of Cardiology, Warren Alpert Medical School of Brown University, Providence, RI USA; 5 Department of Cardiology, Imperial College London Diabetes Centre, Abu Dhabi, United Arab Emirates

**Keywords:** Diabetes mellitus, Sodium–glucose transport protein 2 inhibitor, Glucagon-like peptide-1 receptor agonist, Cardiovascular, Meta-analysis, Observational studies

## Abstract

**Background:**

Randomized controlled trials and real-world studies suggest that combination therapy with sodium–glucose transport protein 2 inhibitors (SGLT2is) and glucagon-like peptide-1 receptor agonists (GLP-1RAs) is associated with improvement in fasting plasma glucose (FPG), glycated hemoglobin (HbA1c), systolic blood pressure (SBP), body mass index (BMI), and total cholesterol levels. However, a systematic review of available real-world evidence may facilitate clinical decision-making in the real-world scenario. This meta-analysis assessed the safety and effectiveness of combinations of SGLT2is + GLP-1RAs with a focus on their cardioprotective effects along with glucose-lowering ability in patients with type 2 diabetes mellitus (T2DM) in a real-world setting.

**Methods:**

Electronic searches were performed in the PubMed/MEDLINE, PROQuest, Scopus, CINAHL, and Google Scholar databases. Qualitative analyses and meta-analyses were performed using the Joanna Briggs Institute SUMARI software package and Review Manager v5.4, respectively.

**Results:**

The initial database search yielded 1445 articles; of these, 13 were included in this study. The analyses indicated that SGLT2is + GLP-1RAs combinations were associated with significantly lower all-cause mortality when compared with individual therapies (odds ratio [95% confidence interval [CI] 0.49 [0.41, 0.60]; p < 0.00001). Significant reductions in BMI (− 1.71 [− 2.74, − 0.67]; p = 0.001), SBP (− 6.35 [− 10.17, − 2.53]; p = 0.001), HbA1c levels (− 1.48 [− 1.75, − 1.21]; p < 0.00001), and FPG (− 2.27 [− 2.78, − 1.76]; p < 0.00001) were associated with the simultaneous administration of the combination. Changes in total cholesterol levels and differences between simultaneous and sequential combination therapies for this outcome were not significant.

**Conclusion:**

This systematic review and meta-analysis based on real-world data suggests that the combination of SGLT2is + GLP-1RAs is associated with lower all-cause mortality and favorable improvements in cardiovascular, renal, and glycemic measurements. The findings drive a call-to–action to incorporate this combination early and simultaneously in managing T2DM patients and achieve potential cardiovascular benefits and renal protection.

**Graphical Abstract:**

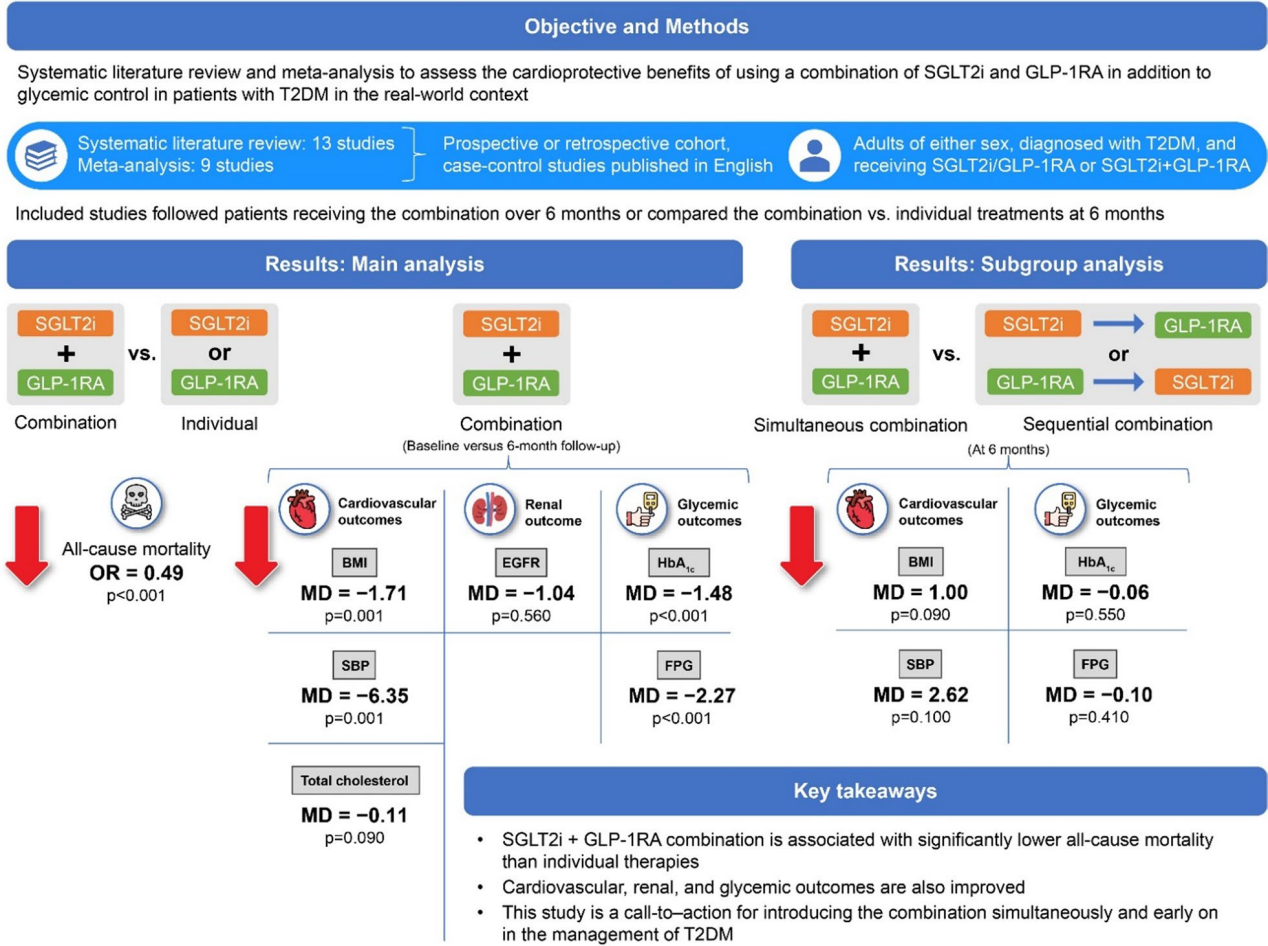

**Supplementary Information:**

The online version contains supplementary material available at 10.1186/s12933-024-02192-4.

## Introduction

Diabetes is a significant predisposing factor for microvascular and macrovascular complications with cardiovascular events 2–3 times more likely to occur in patients with diabetes than in those without diabetes [1]. Conventionally, the management of type 2 diabetes mellitus (T2DM) has been glucocentric rather than focusing on reducing cardiovascular events [2]. However, over the last few years, sodium–glucose transport protein 2 inhibitors (SGLT2is) and glucagon-like peptide-1 receptor agonists (GLP-1RAs) have been shown to reduce the risk of all-cause mortality, cardiovascular mortality, and kidney failure [3]. Recent meta-analyses of the major SGLT2i cardiovascular outcome trials (CVOTs) reported a reduced risk of all-cause mortality and major adverse cardiovascular events (MACE) in T2DM patients using SGLT2i [4, 5]. Similarly, recent meta-analyses of GLP-1 CVOTs reported a reduction in MACE, relative risk of CV deaths, and all-cause mortality [6, 7]. Like SGLT2is [8, 9], the CV effects of GLP-1RAs are reported to be independent of glucose reduction as shown in the multicenter, double-blind, placebo-controlled SELECT study (N = 17,604), in which the GLP-1RA semaglutide significantly reduced the incidence of cardiovascular mortality and that of non-fatal myocardial infarction as well as non-fatal stroke when compared to the placebo (6.5% vs. 8.0%, p < 0.001) in non-diabetes patients with preexisting cardiovascular disease and obesity [10]. While the exact mechanism of action of these two classes of drugs on reducing mortality is still being investigated, several meta-analyses have shown significantly reduced glycated hemoglobin (HbA1c), body weight, body mass index (BMI), systolic blood pressure (SBP), and low-density lipoprotein cholesterol (LDL-C) when either of these drugs was used individually. However, the reduction in these parameters was more significant when the two classes were used in combination with minimal safety concerns resulting in clinical guidelines recommending their use in T2DM patients with established atherosclerotic cardiovascular disease (ASCVD) or with multiple CV risk factors without ASCVD irrespective of the HbA1c level or use of other glucose-lowering medications [7, 11–16]. There has been no direct evidence regarding the effects of the combination of SGLT2is and GLP-1RAs on mortality and other cardiovascular outcomes because of a lack of randomized controlled trials (RCTs) comparing combination versus individual therapies. However, a retrospective study and a subsequent meta-analysis reported significantly reduced risks of MACE, cardiovascular mortality, hypertensive heart failure, and all-cause mortality compared with SGLT2i/GLP1RA monotherapy [17, 18].

To date, there has been no meta-analysis conducted to assess the effect of SGLT2i and GLP-1 combination therapy in the real-world context where results can differ significantly from results from randomized controlled trials (RCTs). Our meta-analysis of real-world data is focused on the impact of such a combination on all-cause mortality and the management of T2DM patients.

## Review question

What is the effectiveness and safety of combinations of SGLT2is + GLP-1RAs in the management of T2DM among adults?

## Methods

This systematic literature review (SLR) was conducted as per the Meta-analysis Of Observational Studies in Epidemiology (MOOSE) reporting guidelines. The protocol was registered with the International Prospective Register of Systematic Reviews PROSPERO (registration number: CRD42023434707).

### Inclusion criteria

This SLR considered observational real-world studies (prospective cohort, retrospective cohort, and case–control studies published in English) that evaluated the effectiveness and safety of combination therapies of SGLT2is and GLP-1RAs in the management of T2DM in adults (≥ 18 years), irrespective of sex, race, ethnicity, or nationality. Systematic reviews, clinical trials, conference abstracts, case series, and case reports were excluded from the analysis. The classification of patients as having T2DM and selection for treatment were determined by the authors of each study included in this SLR. All included studies either followed patients receiving the combination treatment and compared baseline values with those at the end of the follow-up (at least 6 months) or compared the combination treatment against individual treatments at the 6-month follow-up.

### Search strategy

A broad search of MEDLINE (PubMed) was initially undertaken to identify related articles. The index terms derived were then used to develop the search strategy for the PubMed, PROQuest, Scopus, Cumulative Index to Nursing and Allied Health Literature (CINAHL), and Google Scholar (first 100 articles) databases (Additional file 1: Table S1). The search was performed from inception until May 2023. Citation screening using backward and forward citations of included studies was additionally performed. Only those studies published in English were included; no restrictions on publication dates were set on any search.

### Selection of studies

All the citations identified after the search were collated on SR-Accelerator [19], and duplicates were removed. The screening of titles along with abstracts was carried out by two independent authors (AA and HS) as per the review inclusion criteria. Subsequently, when the full texts were screened, articles were closely analyzed for compliance with the inclusion and exclusion criteria in accordance with the Preferred Reporting Items for Systematic Reviews and Meta-analyses (PRISMA) flow diagram. Additionally, a literature mapping was performed using Litmaps® to indicate the relationship among included articles using their citations.

The included studies were assessed for quality using the Joanna Briggs Institute (JBI) Critical Appraisal Checklist for Cohort Studies [20]. Each study was critically appraised by two independent reviewers (AA and HS) based on criteria such as the similarity of study groups, reliability and validity of exposure measurement, identification and handling of confounding factors, freedom from the outcome (at the start of the study), outcome measurement, follow-up time, completeness of follow-up, and appropriateness of statistical analyses. All disagreements regarding appraisals were resolved through discussions. Details are provided in Additional file 2.

All the studies included in this SLR underwent data extraction by two independent authors (AA and HS) with piloted data extraction sheets. Details of the data extraction process are presented in Additional file 2.

### Study outcomes

The outcomes of interest were all-cause mortality, cardiovascular risk factors (BMI, SBP, and total cholesterol), renal outcomes (eGFR and albuminuria), and glycemic outcomes (HbA1c and FPG). Adverse events were also analyzed qualitatively. The term ‘baseline” represented the time when the patients initiated treatment with either the combination or individual treatments, and this definition was consistent across the included studies.

### Subgroup analysis

A subgroup analysis was performed based on whether the patients received the combination simultaneously and had not received either SGLT2is or GLP-1RAs prior to baseline and whether the patients received the combination sequentially, i.e., they were already receiving either SGLT2i or GLP-1RA and the other drug was introduced. The two sequential combination therapy subgroups included patients who were receiving SGLT2i with GLP-1RA added on and patients who were receiving GLP-1RA with SGLT2i added on.

### Synthesis of data

All the extracted data were pooled with the help of a statistical meta-analysis model in the Review Manager v5.4 software (RevMan, Cochrane Collaboration software). For continuous data, effect sizes were presented as weighted (or standardized) final mean differences and their 95% confidence intervals (CIs); for dichotomous data, these were presented as odds ratios and 95% CIs. The *I*^*2*^ statistic for heterogeneity among the included studies was calculated, wherein *I*^*2*^ values of < 30%, 30%–59%, 60%–90%, and > 90% corresponded to low, moderate, substantial, and high heterogeneity, respectively. All analyses were carried out using a random-effects model.

If statistical pooling was not possible, the findings were presented as a narrative, considering the population characteristics, study design, data source, and assessment of the outcome measure.

### Assessing certainty in the findings

Certainties in the quality of the evidence and the estimated effects of the results in this SLR were assessed as per the Grading of Recommendations, Assessment, Development and Evaluation (GRADE) [21]. The findings were summarized using the GRADEPro GDT v.4 software (McMaster University, ON, Canada), and include all the results on all-cause mortality and changes in HbA1c, FPG, BMI, SBP, GFR, and total cholesterol levels.

## Results

### Search results

**Fig. 1:**
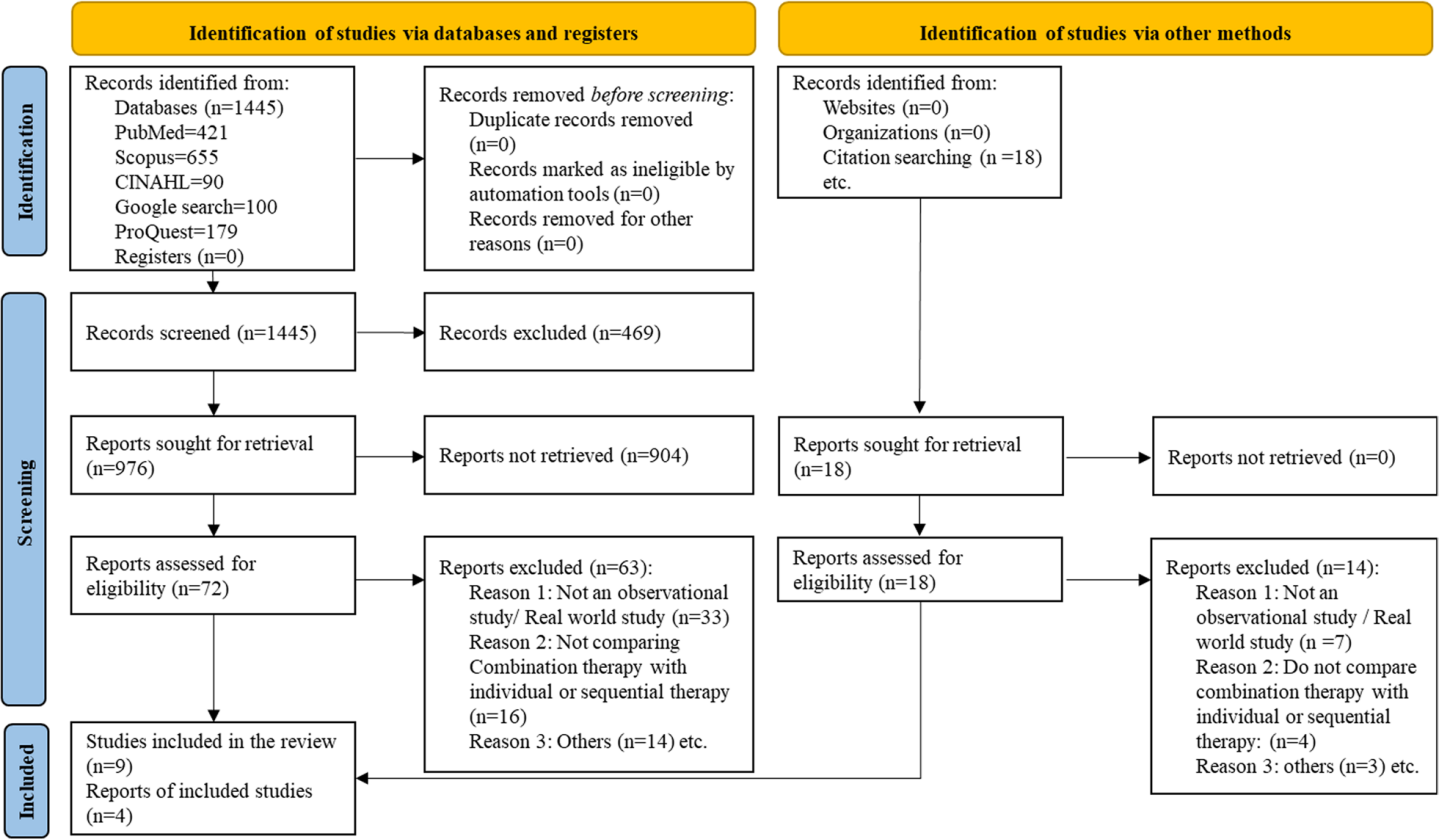
PRISMA flowchart

Of the 1445 articles obtained in the initial database search, 976 were identified based on their titles and abstracts. Manual screening of grey literature resulted in an addition of 18 articles. The full texts of 90 articles were retrieved, 77 studies were excluded (Additional file 1: Table S2), and 13 were included in the study [22–34]. Figure 1 shows the PRISMA flowchart.

**Fig. 2:**
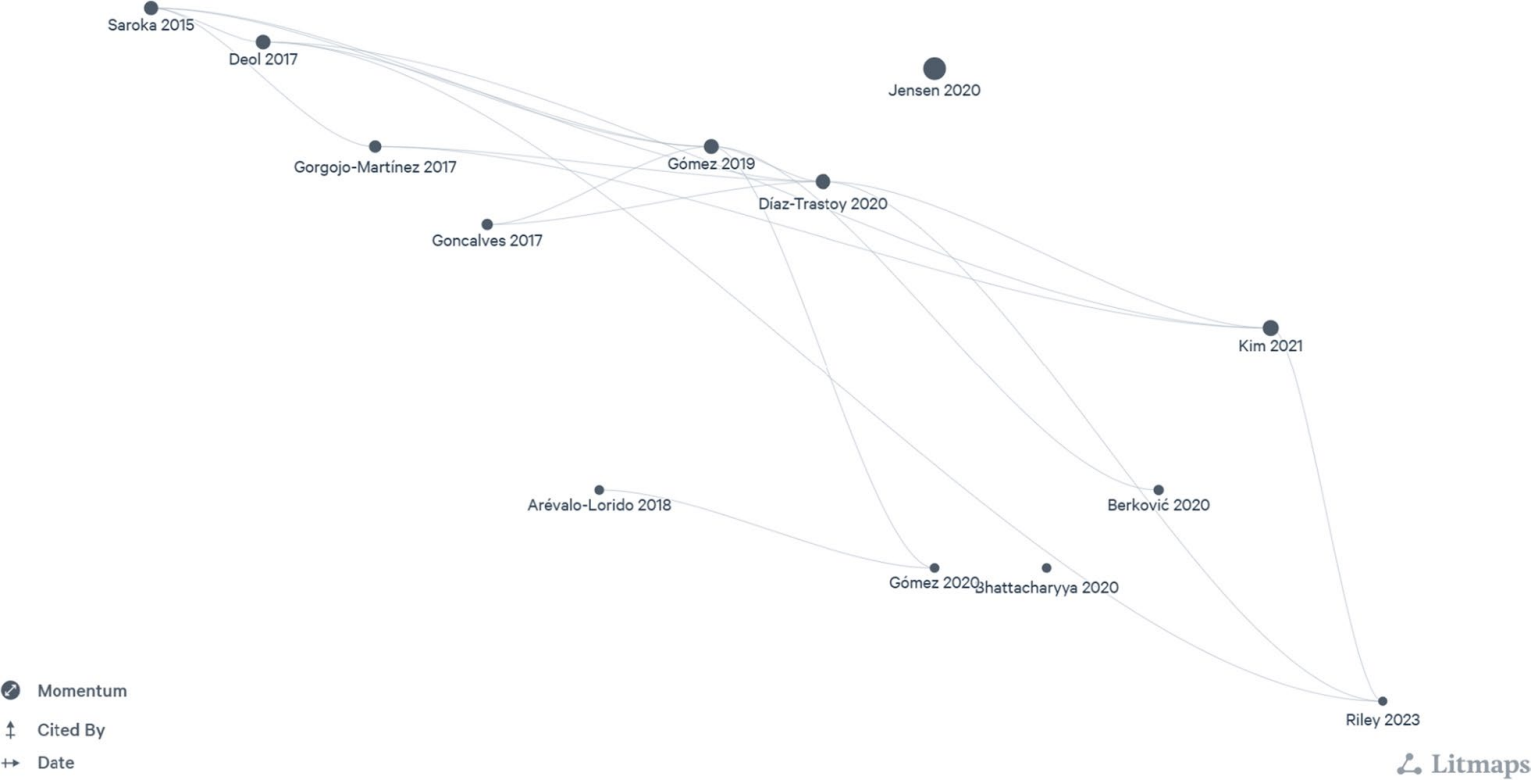
Literature mapping of the included studies (Litmaps®)

Thirteen studies were critically appraised; following this, data were extracted from these studies and analyzed. The literature mapping of the studies included in this SLR is shown in Fig. 2. Quantitative meta-analysis was performed using 8 studies [23, 26–31, 33]. The remaining 5 studies were considered to be ineligible for inclusion in this meta-analysis as mean values were not available (n = 1), follow-up duration was less than 6 months (n = 2), and only differences in outcomes were provided instead of absolute values (n = 2) (Additional file 1: Table S3).

Each point on the map represents one included study in the systematic literature review. The size of each point is a function of the “momentum” of the study, which is calculated based on the “cited by” count and weighted by recency. The most recent articles appear on the right-hand side of the map and the most cited articles appear at the top, so recent popular studies appear in the top right-hand corner.

Ten studies were assessed to be of moderate-to–low quality (score 6–8), and three studies were of low quality (score ≤5) (Table 1). Low-quality studies typically had issues in their methodology, such as unclear or inadequate management of confounding factors, unreliable or invalid outcome measurements, incomplete follow-up without adequate exploration of reasons, and inappropriate or unclear use of statistical analysis. These deficiencies suggest potential biases in the studies, which could affect the trustworthiness of their findings and limit their contributions to evidence-based practice.

**Table 1:** Critical appraisal for methodological quality

Author name and year	Were the two groups similar and recruited from the same population?	Were the exposures measured similarly to assign people to both exposed and unexposed groups?	Was the exposure measured in a valid and reliable way?	Were confounding factors identified?	Were strategies to deal with confounding factors stated?	Were the groups/participants free of the outcome at the start of the study (or at the moment of exposure)?	Were the outcomes measured in a valid and reliable way?	Was the follow-up time reported and sufficient to be long enough for outcomes to occur?	Was follow-up complete, and if not, were the reasons for loss to follow-up described and explored?	Were strategies to address incomplete follow-up utilized?	Was appropriate statistical analysis used?	Score
Berkovic et al. 2020	✓	✓	✓	✓	✓	N/A	✓	✓	✓	N/A	✓	9
Gorgojo-Martínez et al. 2017	✓	✓	✓	✗	?	N/A	✓	✓	✓	N/A	✓	7
Kim et al. 2022	✓	✓	✓	✓	✓	N/A	✓	✓	✓	N/A	✓	9
Deol et al. 2017	✓	✓	✓	✓	✓	N/A	✓	✓	✓	N/A	✓	9
Carretero-Gómez et al. 2019	✓	✓	✓	✓	✓	N/A	✓	✓	✓	N/A	?	8
Arevalo et al. 2018	?	✓	✓	✗	✗	N/A	✓	✓	✓	N/A	?	5
Diaz et al. 2020	✓	✓	✓	✓	✓	N/A	✓	?	?	N/A	?	6
Carretero-Gómez et al. 2020	✓	✓	✓	?	?	N/A	✓	✓	✓	N/A	✓	7
Riley et al. 2023	✓	✓	✓	✓	?	N/A	✓	✗	✗	N/A	?	5
Goncalves et al. 2017	✓	✓	?	✓	✗	N/A	?	✓	✓	N/A	✗	5
Bhattacharyya et al. 2020	✓	✓	✓	✓	✗	N/A	✓	✓	✓	N/A	✗	7
Saroka et al. 2015	✓	✓	?	✓	✗	✓	✓	✓	✓	N/A	✓	8
Jensen et al. 2020	?	✗	✓	✓	✓	N/A	✓	✓	✓	N/A	✓	7

✓: Yes; ✗: No; ?: Unclear; N/A: Not applicable

Given that meta-analysis and subgroup analyses included a relatively small number of studies, inadequate data were available to provide a statistical estimate of publication bias.

### Qualitative analysis (systematic review)

All the studies included in this SLR are real-world observational studies published during 2015–2023, had sample sizes ranging from 15 to 2.2 million, and a follow-up ranging from 3 months to 20 years (Table 2). Patients in the included studies were 49.5–70.4 years old, and 43.6%–65.5% were males (Table 3). Prior comorbidities included cardiovascular disease, hypertension, hyperlipidemia, and obesity. Patients in the included studies reported the concomitant use of other antidiabetic drugs such as metformin, sulfonylureas, and insulin, among others (Table 4).

**Table 2:** Characteristics of the included studies

Study	Country	Participant count	Cohorts, n	Follow-up time point (s)	Study design	Included in the meta-analysis (Y/N)
Arévalo et al. [22]	Spain	17	Simultaneous SGLT2i + GLP-1RA (n = 2) Sequential GLP-1RA + SGLT2i add-on (n = 10) Sequential SGLT2i + GLP-1RA add-on (n = 5)	6 months	Multicenter, prospective cohort study	N
Berkovic et al. [23]	Croatia	200	Simultaneous SGLT2i + GLP-1RA (n = 76) Sequential GLP-1RA + SGLT2i add-on (n = 76) Sequential SGLT2i + GLP-1RA add-on (n = 48)	6 months 12 months	Retrospective and prospective, multicenter, observational cohort study	Y
Bhattacharyya et al. [24]	India	15	GLP-1RA (dulaglutide) + SGLT2i + metformin with or without insulin	3 months	Retrospective, single-center, observational study	N
Carretero Gómez et al. [27]	Brazil	113	Simultaneous SGLT2i + GLP-1RA (n = 30) Sequential GLP-1RA + SGLT2i add-on (n = 59) Sequential SGLT2i + GLP-1RA add-on (n = 24)	3 months 6 months	Prospective, observational, multicenter cohort study	Y
Carretero Gómez et al. [28]	Brazil	178	Simultaneous SGLT2i + GLP-1RA (n = 52) Sequential GLP-1RA + SGLT2i add-on (n = 76) Sequential SGLT2i + GLP-1RA add-on (n = 50)	6 months	Prospective, observational, multicenter cohort study	Y
Deol et al. [25]	UK	79	Sequential GLP-1RA + SGLT2i add-on (n = 79)	3–6 months	Retrospective, single-center, observational cohort study	N
Díaz-Trastoy et al. [26]	Spain	212	Simultaneous SGLT2i + GLP-1RA (n = 38) Sequential GLP-1RA + SGLT2i add-on (n = 85) Sequential SGLT2i + GLP-1RA add-on (n = 89)	5.5 months 16.4 months	Retrospective, single-center, observational cohort study	Y
Goncalves et al. [29]	USA	79	Simultaneous SGLT2i + GLP-1RA (n = 33) Sequential GLP-1RA + SGLT2i add-on (n = 46)	Simultaneous SGLT2i + GLP-1RA: 14 months Sequential GLP-1RA + SGLT2i add-on: 18 months	Retrospective, single-center, observational cohort study	Y
Gorgojo-Martínez et al. [32]	Spain	213	Sequential GLP-1RA + SGLT2i (dapagliflozin) add-on (n = 109) SGLT2i (dapagliflozin) (n = 103)	6 months 12 months	Retrospective, single-center, observational cohort study	N
Jensen et al. [30]	Denmark	66,807	SGLT2i (n = 3405) GLP-1RA (n = 11,436) SGLT2i + GLP-1RA (n = 1823)	20 years	Time-to–event cohort study based on the Danish National Registry	Y
Kim et al. [31]	Korea	104	SGLT2i + GLP-1RA (n = 104)	6 months 12 months	Retrospective, single-center, observational cohort study	Y
Riley et al. [33]	Global	2.2 million	SGLT2i (n = 1,43,600) GLP-1RA (n = 1,86,841) SGLT2i + GLP-1RA (n = 1,08,504)	5 years	Retrospective, observational cohort study based on inpatient and outpatient electronic medical records from TriNetX	Y
Saroka et al. [34]	USA	75	Sequential GLP-1RA + SGLT2i (canagliflozin) add-on (n = 75)	40 months	Retrospective, pre-post, single-center, observational cohort study	N

*GLP-1RA* Glucagon-like peptide-1 receptor agonist, *SGLT2i* Sodium–glucose transport protein 2 inhibitor, *UK* United Kingdom, *USA* United States of America

**Table 3:** Characteristics of participants in the included studies

Study	Participant count	Males, %	Participant age, years, mean ± SD	Duration of diabetes, years	Baseline HbA1c, mean ± SD, %	Baseline SBP, mean ± SD, mmHg	Baseline BMI, mean ± SD, kg/m^2^	Comorbidities
Arévalo et al. [22]	17	52.9	66.8 ± 8.3 (median)	N/A	8.1 ± 0.8	124 ± 11	30.8 ± 3.8	N/A
Berkovic et al. [23]	200	N/A	62.1 ± 9.5	11.7 ± 6.2	8.32 ± 1.26	N/A	39.41 ± 5.49	N/A
Bhattacharyya et al. [24]	15	60.0	49.5 ± 9.3	7.6 ± 1.2	8.61 ± 1.41	129.87 ± 4.81	32.27 ± 4.67	N/A
Carretero Gómez et al. [27]	113	65.5	70.4 ± 8.8	N/A	8.04 ± 1.2	136.1 ± 17	36.5 ± 6.6	Hypertension (69.9%), dyslipidemia (82.3%), coronary artery disease (19.5%), heart failure (9.73%)
Carretero Gómez et al. [28]	178	58.6	61.9 ± 10.0	10.0 ± 6.7	8.2 ± 0.9	138.3 ± 16.9	36.2 ± 10	Hypertension (80.9%), dyslipidemia (81.4%), coronary artery disease (7.9%)
Deol et al. [25]	79	51.1	57.4 ± 7.8	13.1 ± 7.2	8.8 ± 1.47	134 ± 16	38.4 ± 6.3	N/A
Díaz-Trastoy et al. [26]	212	47.6	61.5 ± 9.6	12.3 ± 7.0	8.4 ± 1.2	137.4 ± 17.9	37.7 ± 8.1	Hypertension (51.1%), retinopathy (14.6%), nephropathy (10.4%), peripheral neuropathy (3.3%), autonomic neuropathy (1.9%), cardiovascular disease (11.8%), cerebrovascular disease (1.4%), heart failure (0.5%), pulmonary edema (0.9%)
Goncalves et al. [29]	79	Sequential GLP-1RA + SGLT2i add-on: 50.0 Simultaneous SGLT2i + GLP-1RA: 48.0	Sequential GLP-1RA + SGLT2i add-on: Median 60.5 ± 7.1 Simultaneous SGLT2i + GLP-1RA: Median 51.0 ± 10.0	Simultaneous SGLT2i + GLP-1RA: 9.3 ± 6.0 Sequential GLP-1RA + SGLT2i add-on: 11.0 ± 6.0	Sequential GLP-1RA + SGLT2i add-on: 8.9 ± 1.3 Simultaneous SGLT2i + GLP-1RA: 9.1 ± 1.4	Sequential GLP-1RA + SGLT2i add-on: 133 ± 17 Simultaneous SGLT2i + GLP-1RA: 135 ± 16	Sequential GLP-1RA + SGLT2i add-on: 37.2 ± 5.2 Simultaneous SGLT2i + GLP-1RA: 39.2 ± 8.2	N/A
Gorgojo-Martínez et al. [32]	213	Sequential GLP-1RA + SGLT2i (dapagliflozin) add-on: 59.6 SGLT2i (dapagliflozin): 47.1	Sequential GLP-1RA + SGLT2i (dapagliflozin) add-on: 59.1 ± 10.7 SGLT2i (dapagliflozin): 59.7 ± 10.8	Sequential GLP-1RA + SGLT2i (dapagliflozin) add-on: 12.4 SGLT2i (dapagliflozin): 9.1	Sequential GLP-1RA + SGLT2i (dapagliflozin) add-on: 7.4 ± 1.3 SGLT2i (dapagliflozin): 7.3 ± 1.3	Sequential GLP-1RA + SGLT2i (dapagliflozin) add-on: 139.5 ± 15.5 SGLT2i (dapagliflozin): 139.3 ± 13.5	Sequential GLP-1RA + SGLT2i (dapagliflozin) add-on: 35.0 ± 4.2 SGLT2i (dapagliflozin): 35.9 ± 8.2	Sequential GLP-1RA + SGLT2i (dapagliflozin) add-on: Hypertension (82.6%), hypercholesterolemia (93.6%), hypertriglyceridemia (63.3%), current smoker (17.4%), diabetic retinopathy (15.6%), diabetic renal disease (29.4%), diabetic neuropathy (11.1%), coronary artery disease (13.8%), stroke (5.5%), peripheral artery disease (12.8%)
Jensen et al. [30]	66,807	SGLT2i: 63.8 GLP-1RA: 52.1 SGLT2i + GLP-1RA: 64.8	SGLT2i: 59.0 ± 12.0 GLP-1RA: 58.0 ± 12.0 SGLT2i + GLP-1RA: 57.0 ± 11.0	SGLT2i: 6.0 ± 5.0 GLP-1RA: 7.0 ± 5.0 SGLT2i + GLP-1RA: 9.0 ± 5.0				N/A
Kim et al. [31]	104	48.7	51.1 ± 10.6	N/A	9.02 ± 1.39	132.78 ± 16.49	28.78 ± 4.28	N/A
Riley et al. [33]	2.2 million	SGLT2i: 59.6 GLP-1RA: 43.6 SGLT2i + GLP-1RA: 50.9	SGLT2i: 62.8 ± 12.2 GLP-1RA: 58.7 ± 13.0 SGLT2i + GLP-1RA: 58.7 ± 11.5	N/A				N/A
Saroka et al. [34]	75	56.0	58.0 ± 8.6	13.8 ± 6.4	7.94 ± 0.69	121 ± 11	39.4 ± 9.4	Hypertension (90.7%), dyslipidemia (94.7%), microvascular disease (32.0%), macrovascular disease (22.7%)

*BMI* Body mass index, *GLP-1RA* glucagon-like peptide-1 receptor agonist, *HbA1c* glycated hemoglobin, *N/A* not available, *SBP* systolic blood pressure, *SD* standard deviation, *SGLT2i* sodium–glucose transport protein 2 inhibitor

**Table 4:** Antidiabetic drugs used by patients in the included studies

Study	Number of patients (N)	SGLT2i, %	GLP-1RA, %	Other concurrent antidiabetic medications, %
Arévalo et al. [22]	17	Dapagliflozin: 47.1% Canagliflozin: 29.4% Empagliflozin: 23.5%	Liraglutide: 82.3% Dulaglutide: 11.8% Albiglutide: 5.9%	N/A
Berkovic et al. [23]	200			*SGLT2i* Insulin: 32.4% Metformin: 94.1% SU: 11.8% Other: 8.8% *GLP-1RA* Insulin: 45.2% Metformin: 85.5% SU: 19.4% Other: 16.1% *SGLT2i + GLP-1A* Insulin: 26.2% Metformin: 78.6% SU: 14.3% Other: 19.0%
Bhattacharyya et al. [24]	15	Canagliflozin, empagliflozin, or dapagliflozin: 100%	Dulaglutide: 100%	Sitagliptin/vildagliptin/linagliptin + glimepiride: 100% Insulin (8.35 ± 0.45 U): 26.7%
Carretero Gómez et al. [27]	113	Canagliflozin: 58.4% Dapagliflozin: 26.5% Empagliflozin: 15.04%	Liraglutide: 52.2% Dulaglutide: 29.2% Exenatide LAR: 8.84% Lixisenatide: 5.31% Albiglutide: 4.42%	Insulin (39.4 ± 19.5 U): 46%
Carretero Gómez et al. [28]	178	Canagliflozin: 46.6% Dapagliflozin: 29.8% Empagliflozin: 23.6%	Liraglutide: 52.2% Dulaglutide: 33.1% Exenatide LAR: 7.3% Lixisenatide: 4.5% Albiglutide: 2.8%	N/A
Deol et al. [25]	79	Dapagliflozin: 97.3% Canagliflozin: 2.7%		Insulin (89.0 ± 51.0 U): 62.2% Metformin: 78.4%
Díaz-Trastoy et al. [26]	212	Dapagliflozin: 45.8% Empagliflozin: 35.8% Canagliflozin: 18.4%	Dulaglutide: 52.4% Liraglutide: 26.9% Exenatide LAR: 17% Lixisenatide: 3.8%	Metformin: 86.8% Sulfonylureas: 10.8% DPP-4 inhibitors: 1.9% Pioglitazone: 0.9% Repaglinide: 0.9% Insulin (59.3 ± 38.7 U): 41%
Goncalves et al. [29]	79	Canagliflozin: 75.0% Empagliflozin: 35.0%	Liraglutide: 66%	N/A
Gorgojo-Martínez et al. [32]	213	Dapagliflozin: 100%	Liraglutide: 72.5% Exenatide (once weekly): 20.2% Exenatide (twice daily): 2.8% Lixisenatide: 4.6%	*Sequential GLP-1RA + SGLT2i add-on* Metformin: 96.3% SU: 13.8% Glitazones: 7.3% Insulin (56.6 ± 41.6 U): 48.6% *SGLT2i* Metformin: 83.7% SU: 19.2% DPP-4 inhibitors: 41.3% Insulin (45.3 ± 30.5 U): 35.6%
Jensen et al. [30]	66,807	Dapagliflozin: 49.7% Canagliflozin: 3.4% Empagliflozin: 46.9%	Liraglutide: 96.2% Exenatide: 2.2% Lixisenatide: 0.1% Dulaglutide: 1.5%	Metformin: 100%
Kim et al. [31]	104	Dapagliflozin or empagliflozin: 100%	Dulaglutide: 100%	Metformin: 98.1% SU: 81.7% Insulin (53.38 ± 24.57 U): 10.6%
Riley et al. [33]	2.2 million	N/A	N/A	N/A
Saroka et al. [34]	75	Canagliflozin: 100%	Liraglutide: 62.7% Exenatide (once weekly): 25.3% Exenatide (twice daily): 12.0%	Metformin: 78.7% Insulin: 60% Thiazolidinediones: 25.3% SU: 16% Colesevelam: 4% DPP-4 inhibitor: 2.7% Meglitinide: 1.3%

*DPP* Dipeptidyl peptidase, *GLP-1RA* glucagon-like peptide-1 receptor agonist, *N/A* not available, *SGLT2i* sodium–glucose transport protein 2 inhibitor, *SU* Sulfonylurea

Genital infections, urinary tract infections, abdominal pain, nausea, bloated abdomen, diarrhea, polyuria, asthenia, yeast infections, dry mouth, and hypotension were among the commonly reported adverse events in the included studies. In the study by Carretero-Gomez et al. [27], symptomatic hypoglycemia was reported in < 10% of the patients and one death due to subarachnoid hemorrhage was reported. Treatment with SGLT2i was discontinued due to genital mycotic infection, worsening of renal function, leg amputation, and bariatric surgery. Treatment with GLP-1RA was discontinued due to gastrointestinal effects, insulin intensification, and worsening of renal function. In the study by Kim et al. [31], gastrointestinal adverse effects were commonly reported after 3 months, but their incidence was reduced at later time points. Mild hypoglycemia was also reported in < 5% of the patients. Major adverse effects such as ketoacidosis, pancreatitis, fractures, or acute renal failure were not reported.

### Quantitative analysis (meta-analysis)

#### All-cause mortality

**Fig. 3:**
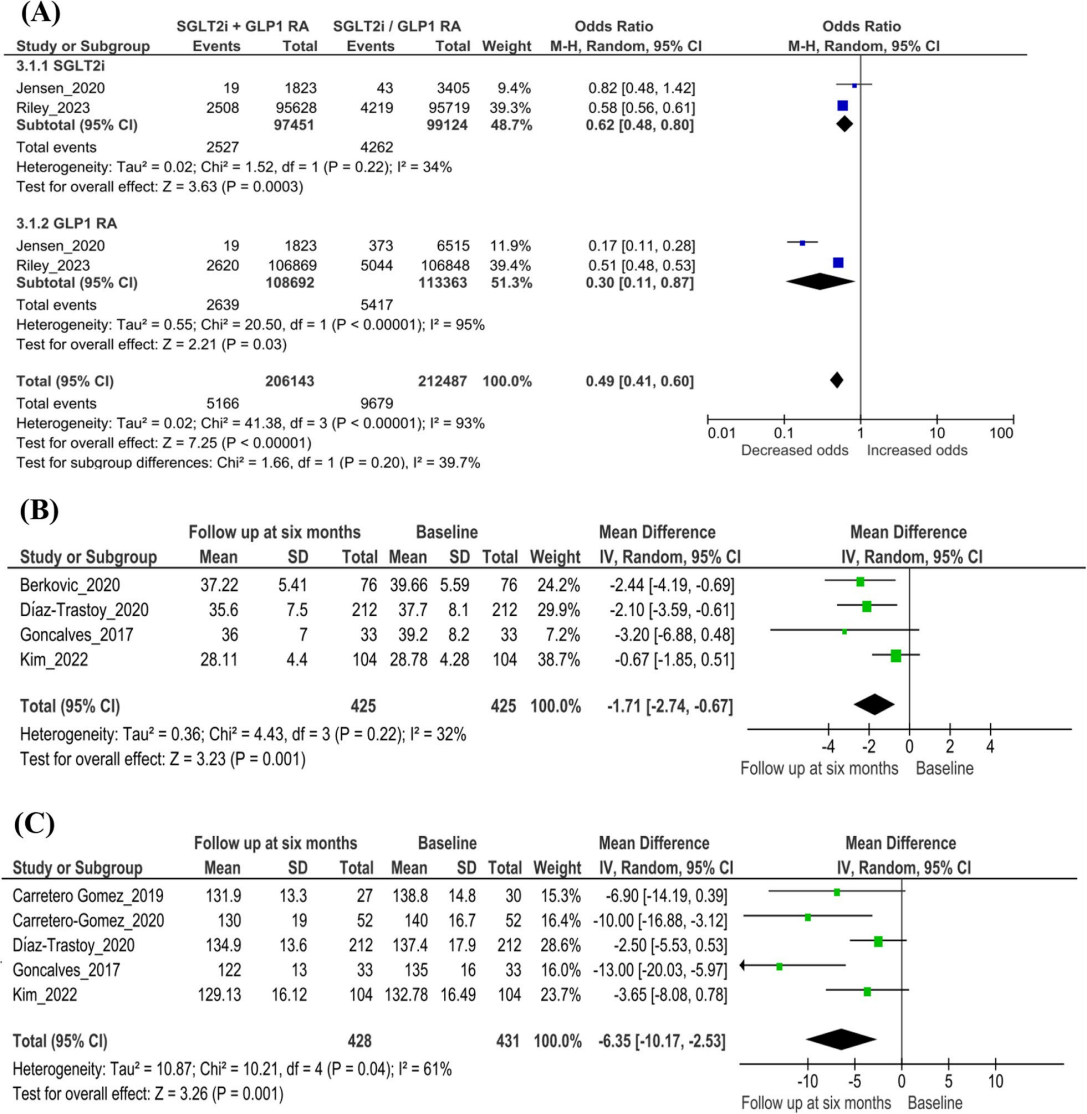

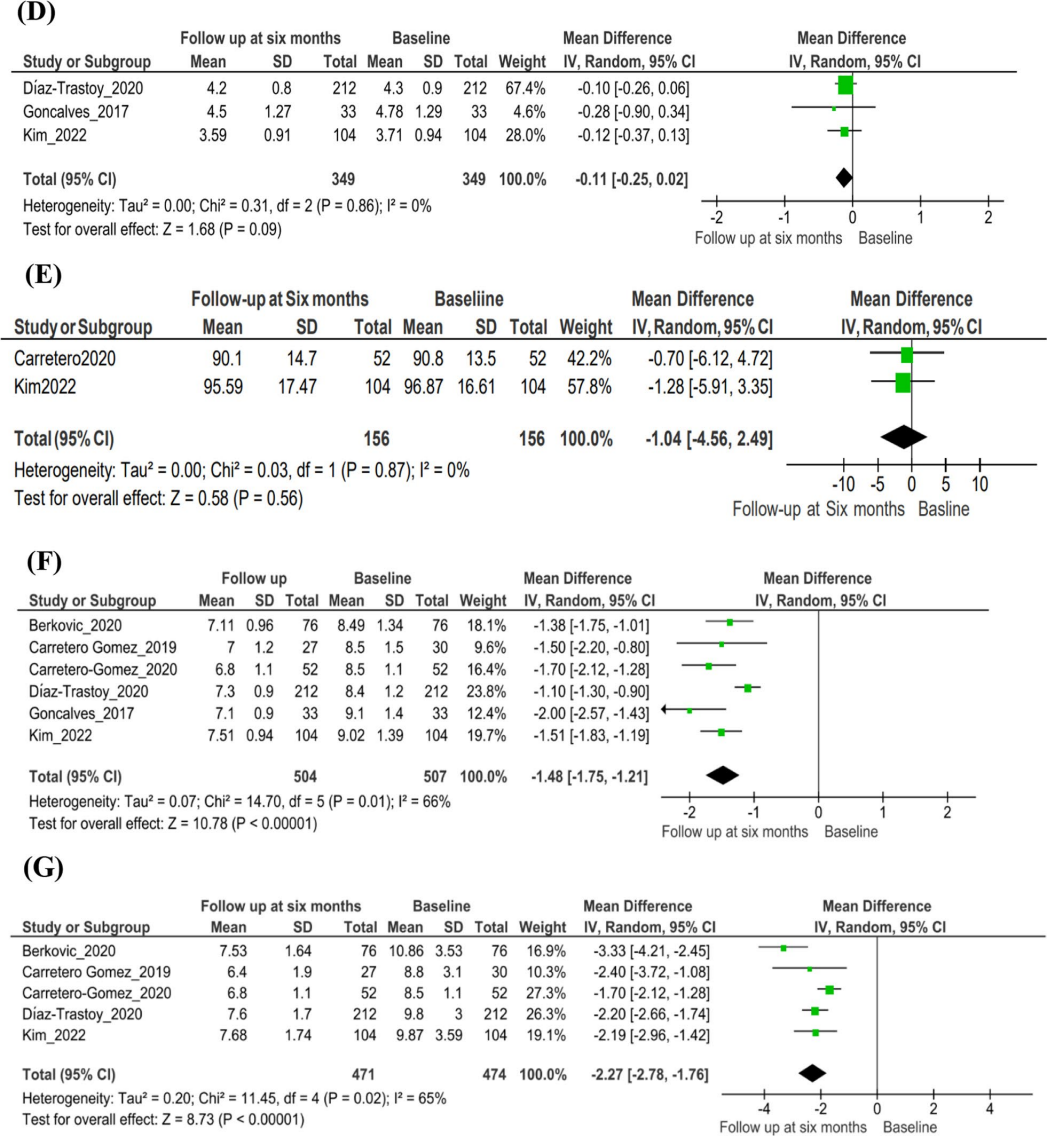
Findings from the main analysis. **A** Odds of all-cause mortality with SGLT2i + GLP-1RA combination therapy versus SGLT2i or GLP-1RA therapy. **B** Changes in BMI with combination therapy at the baseline versus the 6-month follow-up. **C** Changes in SBP with combination therapy at the baseline versus the 6-month follow-up. **D** Changes in total cholesterol with combination therapy at the baseline versus the 6-month follow-up. **E** Changes in eGFR with simultaneous combination therapy at the baseline versus the 6-month follow-up. **F** Changes in HbA1c with combination therapy at the baseline versus the 6-month follow-up. **G** Changes in FPG with combination therapy at the baseline versus the 6-month follow-up. *BMI* Body mass index, *CI* confidence interval, *eGFR* estimated glomerular filtration rate, *FPG* fasting plasma glucose, *M–H* Mantel–Haenszel, *GLP-1RA* glucagon-like peptide-1 receptor agonist, *HbA1c* glycated hemoglobin, *IV* importance value, *SBP* systolic blood pressure, *SD* standard deviation, *SGLT2i* Sodium–glucose transport protein 2 inhibitor

The number of all-cause mortality events was significantly lower with the combination therapy than with SGLT2i (p = 0.0003) or GLP-1RA (p = 0.03), with an overall decrease in the odds for all-cause mortality with the combination therapy (n = 2 studies; odds ratio [95% CI] 0.49 [0.41, 0.60]; *I*^*2*^ = 93.0%; p < 0.00001) (Fig. 3A).

#### Cardiovascular risk factors

The combination significantly reduced BMI and SBP at follow-up (BMI: n = 4 studies; mean difference [95% CI] − 1.71 [− 2.74, − 0.67]; *I*^*2*^ = 32%; p = 0.001, Fig. 3B; SBP: n = 5 studies; mean difference [95% CI] − 6.35 [− 10.17, − 2.53]; *I*^*2*^ = 61%; p = 0.001, Fig. 3C). Although there was a decrease in the mean total cholesterol levels at follow-up from baseline with the combination therapy, the difference was not significant (n = 3 studies; mean difference [95% CI] − 0.11 [− 0.25, 0.02]; *I*^*2*^ = 0%; p = 0.09) (Fig. 3D).

#### Renal outcomes

The combination therapy was not associated with significant decreases in eGFR levels at follow-up from baseline (n = 2 studies; mean difference [95% CI]  − 1.04 [− 4.56, 2.49]; *I*^*2*^ = 0%; p = 0.56) (Fig. 3E). Two studies (Carretero-Gomez et al. [28] and Diaz-Trastoy et al. [26]) reported results related to albuminuria. In the study by Carretero-Gomez et al. [28], there was a significant reduction in total urinary albumin-to–creatinine ratio (UACR; − 15.14 mg/g; p < 0.0001) and macroalbuminuria (UACR > 30 mg/g; − 63.18 mg/g; p < 0.0001) at 26 weeks. In the study by Diaz-Trastoy et al. [26], albuminuria data were available for 127 of 212 patients, 10 patients progressed from normoalbuminuria to micro- and macroalbuminuria, and 13 patients showed regression.

#### Glycemic outcomes

Treatment with the SGLT2i + GLP-1RA combination was also associated with improved glycemic control, expressed as a significant decrease in HbA1c and FPG levels at the 6-month follow-up from baseline (HbA1c: n = 7 studies; mean difference [95% CI] − 1.48 [− 1.75, − 1.21]; *I*^*2*^ = 66.0%; p < 0.00001, Fig. 3F; FPG: n = 5 studies; mean difference [95% CI] − 2.27 [− 2.78, − 1.76];* I*^*2*^ = 65.0%; p < 0.00001, Fig. 3G).

### Findings from the subgroup analyses

#### Cardiovascular risk factors

**Fig. 4:**
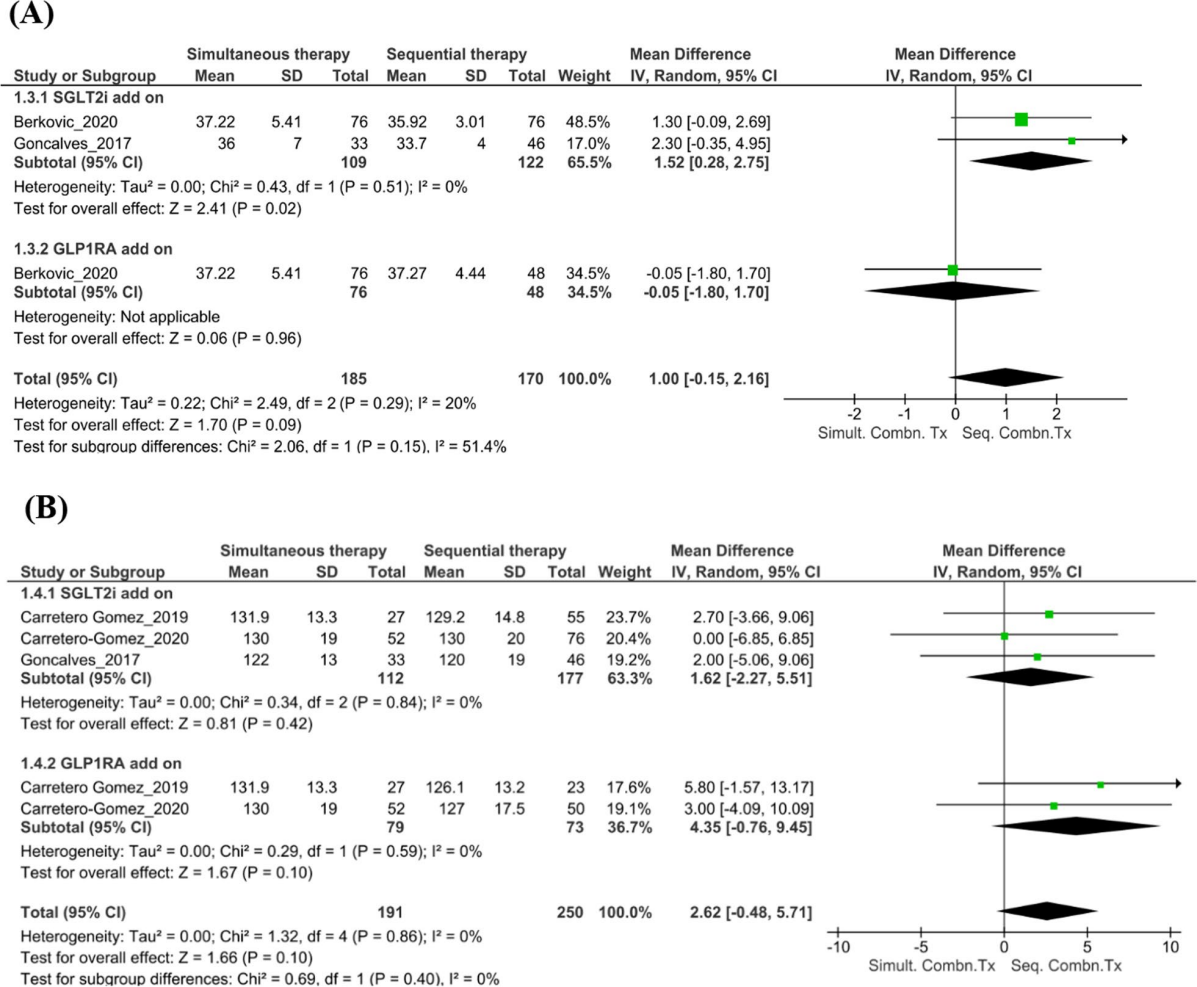

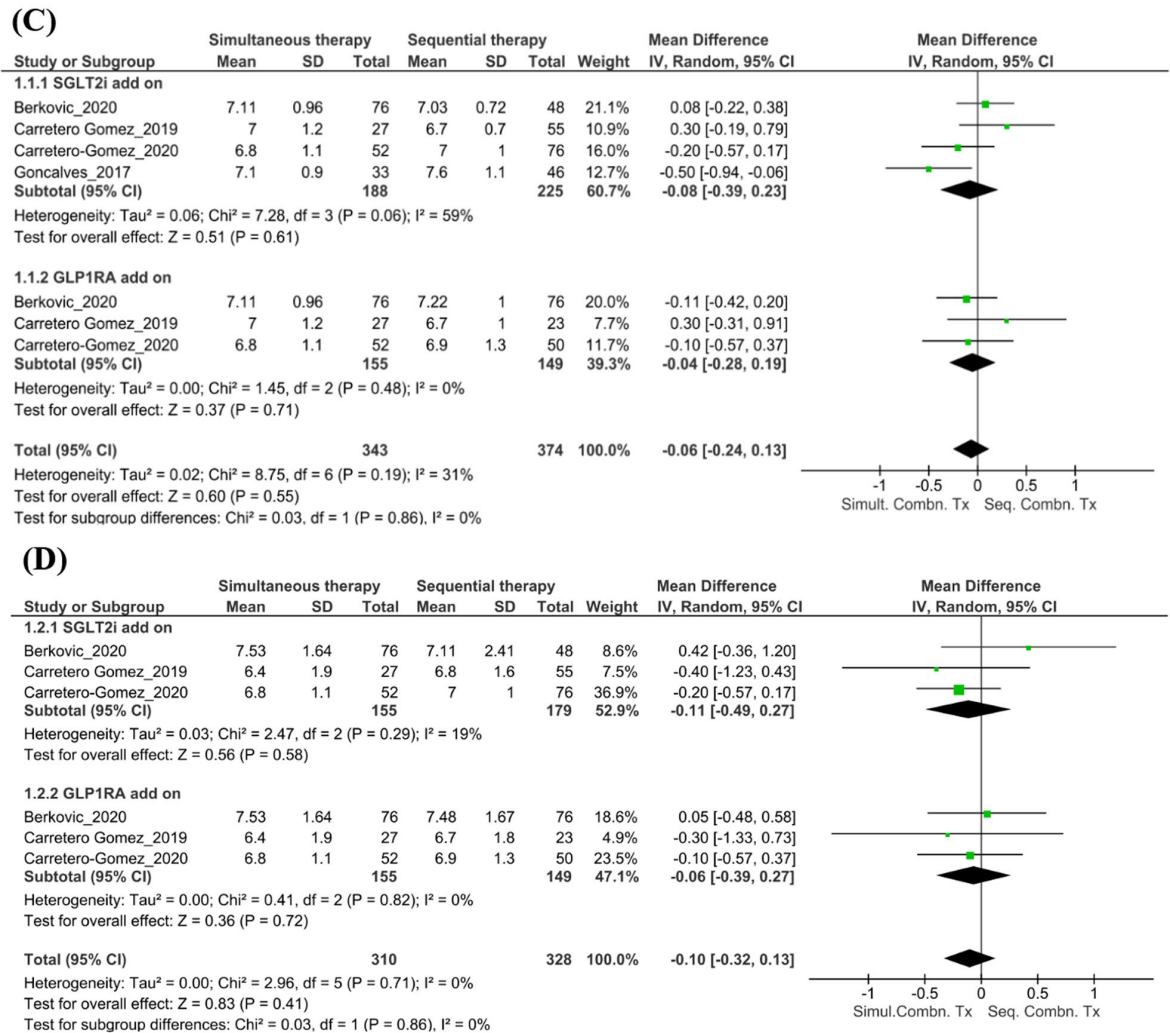
Findings from the subgroup analyses. **A** Changes in BMI with simultaneous versus sequential combination therapy at the 6-month follow-up. **B** Changes in SBP with simultaneous versus sequential combination therapy at the 6-month follow-up. **C** Changes in HbA1c levels with simultaneous versus sequential combination therapy at the 6-month follow-up. **D** Changes in FPG levels with simultaneous versus sequential combination therapy at the 6-month follow-up. *BMI* body mass index, *CI* confidence interval, *FPG* fasting plasma glucose, *GLP-1RA* glucagon-like peptide-1 receptor agonist, *HbA1c* glycated hemoglobin, *IV* importance value, *SBP* systolic blood pressure, *SD* standard deviation, *SGLT2i* sodium–glucose transport protein 2 inhibitor, *Seq. Combn. Tx* sequential combination therapy, *Simult. Combn. Tx* simultaneous combination therapy

The pattern of administration of the combination therapy did not significantly affect BMI at follow-up (n = 2 studies; mean [95% CI] 1.00 [− 0.15, 2.16]; *I*^*2*^ = 20%; p = 0.09, Fig. 4A). However, the reduction in BMI was significant when patients were already on GLP-1RA and received add-on SGLT2i (n = 2 studies; mean [95% CI] 1.52 [0.28, 2.75]; *I*^*2*^ = 0%; p = 0.02). Although there was a reduction in the SBP levels in those on sequential combination therapy compared with those on simultaneous combination therapy at 6 months follow-up, this change was not significant (n = 5 studies; mean [95% CI] 2.62 [− 0.48, 5.71]; *I*^*2*^ = 0%; p = 0.10, Fig. 4B).

#### Renal outcomes

In the study by Carretero-Gomez et al. [28], the extent of reduction in the total UACR was significant and similar when the combination was started simultaneously or when a GLP-1RA was added to ongoing SGLT2i therapy (− 17.19 mg/g and − 16.4 mg/g, respectively; p < 0.0001). A greater reduction in macroalbuminuria was observed when an SGLT2i was added to a GLP-1RA than when a GLP-1RA was added to an SGLT2i (− 116.7 mg/g [n = 24] and − 55.5 mg/g [n = 21]; p < 0.005).

#### Glycemic outcomes

For the subgroup analyses, outcomes at the 6-month follow-up were compared between patients treated with the combination of SGLT2i + GLP-1RA administered simultaneously versus sequentially. Pooled estimates for changes in HbA1c levels and FPG levels showed no significant differences between the simultaneous combination therapy and sequential combination therapy at follow-up (HbA1c: n = 4 studies; mean [95% CI] − 0.06 [− 0.24, 0.13];* I*^*2*^ = 31.0%; p = 0.55, Fig. 4C; FPG: n = 2 studies; mean [95% CI] − 0.10 [− 0.32, 0.13]; *I*^*2*^ = 0%; p = 0.41, Fig. 4D).

Due to the lack of studies comparing simultaneous combination and sequential combination therapies at follow-up, meta-analysis could not be performed for all-cause mortality outcomes, and changes in eGFR and total cholesterol levels.

### GRADE analysis

According to the GRADE analysis (Table 5), the certainty of evidence for the outcomes of all-cause mortality and changes in BMI, SBP, HbA1c, eGFR, and total cholesterol levels was “low.” The certainty of evidence for the outcome of changes in FPG was “very low.”

**Table 5:** Findings of the GRADE analysis

Combination of SGLT2i and GLP-1RA therapy compared to SGLT2i or GLP-1RA therapy for type 2 diabetes
Patient or population: T2DM Intervention^a^: Combination of SGLT2i and GLP-1RA therapy Comparison^a^: SGLT2i or GLP-1RA therapy

Outcome	Anticipated absolute effects (95% CI)	Relative effect (95% CI)	No. of participants (studies)	Certainty of the evidence (GRADE)	Comments
	Risk with combination of SGLT2i and GLP-1RA therapy				
All-cause mortality	22 per 1000 (19 to 27)	RR 0.49 (0.41 to 0.60)	4,18,630 (2 observational studies)	⨁⨁◯◯ Low	Combination of SGLT2i and GLP-1RA therapy may result in a large reduction in all-cause mortality due to T2DM
Change in HbA1c Follow-up: 6 months	MD 1.48 lower (1.75 lower to 1.21 lower)	–	1011 (7 observational studies)	⨁⨁◯◯ Low	Combination of SGLT2i and GLP-1RA therapy may result in a slight (but important) reduction in HbA1C
Change in FPG Follow-up: 6 months	MD 2.27 lower (2.78 lower to 1.76 lower)	–	945 (5 observational studies)	⨁◯◯◯ Very low	Combination of SGLT2i and GLP-1RA therapy may reduce FPG but the evidence is very uncertain
Change in BMI Follow-up: mean 6 months	MD 1.71 lower (2.74 lower to 0.67 lower)	–	850 (4 observational studies)	⨁⨁◯◯ Low	The combination of SGLT2i and GLP-1RA therapy may reduce BMI
Change in SBP Follow-up: 6 months	MD 6.35 lower (10.17 lower to 2.53 lower)	–	859 (5 observational studies)	⨁⨁◯◯ Low	Evidence suggests that the combination of SGLT2i and GLP-1RA therapy results in a large reduction in SBP
Change in eGFR Follow-up: 6 months	MD 1.04 lower (4.56 lower to 2.49 higher)	–	312 (2 observational studies)	⨁⨁◯◯ Low	The combination of SGLT2i and GLP-1RA therapy may reduce eGFR
Change in total cholesterol Follow-up: 6 months	MD 0.11 lower (0.25 lower to 0.02 higher)	–	698 (3 observational studies)	⨁⨁◯◯ Low	The combination of SGLT2i and GLP-1RA therapy may reduce total cholesterol

*BMI* Body mass index, *CI* confidence interval, *eGFR* estimated glomerular filtration rate, *FPG* fasting plasma glucose, *GLP-1RA* glucagon-like peptide-1 receptor agonist, *GRADE* Grading of Recommendations, Assessment, Development and Evaluation, *HbA1c* glycated hemoglobin, *MD* mean difference, *RR* relative risk, *SBP* systolic blood pressure, *SD* standard deviation, *SGLT2i* sodium–glucose transport protein 2 inhibitor, *T2DM* type 2 diabetes mellitus

^a^Intervention and comparator for changes in HbA1c, FPG, BMI, SBP, eGFR, and total cholesterol was the combination of SGLT2i + GLP-1RA administered simultaneously, but MD was calculated based on the difference in the parameters between baseline and follow-up at 6 months

## Discussion

Our meta-analysis of real world data showed a significant reduction in all-cause mortality as well as significant reductions in HbA1c, SBP, and body weight in T2DM patients treated with SGLT2i + GLP-1RA combination compared to either 2 drug class used alone.

Individual use of SGLT2is and GLP-1RAs can significantly improve cardiovascular outcomes and reduce mortality. This association has been reported in several landmark RCTs and meta-analyses [7, 11, 12, 35–41]. Empagliflozin was associated with a reduction in CV mortality, nonfatal MI, or nonfatal stroke, as well as a reduction in all-cause mortality [35–37], whereas DECLARE showed dapagliflozin reduced all-cause mortality in patients with heart failure with reduced ejection fraction (HFrEF) but not in those without HFrEF [39] The CANVAS and CANVAS-R studies on canagliflozin have reported similar benefits [38]. Death from CV causes and all-cause mortality were reduced in participants receiving liraglutide in the LEADER study [42, 43], while the SUSTAIN-6 study demonstrated lower CV deaths in patients receiving subcutaneous semaglutide [44]. Oral semaglutide significantly reduced CV risk factors such as HbA1c, body weight, and SBP with nearly 50% reduction in CV and all-cause mortality in the PIONEER-6 trial [45]. The SOUL study demonstrated the non-inferiority of semaglutide to placebo in terms of CV mortality outcomes [46]. The REWIND study reported a lower incidence of MACE-3 and other CV outcomes [41], whereas, albiglutide reduced CV events driven by a reduction in myocardial infarction with similar CV deaths [40].

In the absence of studies directly comparing SGLT2i/GLP-1RA combination versus either of these being used alone, meta-analyses of CVOTs and observational studies have assessed whether the concurrent use of SGLT2is + GLP-1RAs provides an additional advantage in reducing the incidence of MACE, cardiorenal endpoints, and cardiovascular mortality. A retrospective cohort study evaluating the added benefits of administering GLP-1RAs + SGLT2is in patients with T2DM (N = 5576) showed that the addition of GLP-1RA reduced the risk of composite all-cause mortality, myocardial infarction, and stroke by 67% [17]. However, an analysis of three US claims datasets from 2013 to 2018 for all-cause mortality among patients (N = 12,584) who received add-on SGLT2i while already receiving GLP-1RA therapy showed that although the SGLT2i addition reduced the risk of MACE and hospitalizations related to heart failure, it did not reduce all-cause mortality [47]. In a study by Jensen et al. [30], treatment with metformin + SGLT2is and metformin + GLP-1RAs was associated with a reduced risk of MACE, all-cause mortality, and severe hypoglycemia; however, patients who received triple therapy with SGLT2i + GLP-1RAs + metformin had the lowest risk of all three outcomes.

Although initial metanalyses on SGLT21/GLP-1 combination reporting greater reductions in HbA1c, SBP, and body weight were not powered or did not assess MACE or all-cause mortality, a later meta-analysis of CVOTs using SGLT2i or GLP-1 reported significant reductions in MACE (30%), cardiovascular mortality/hospitalization due to heart failure (31%), and all-cause mortality (57%) compared with monotherapy with either SGLT2i or GLP-1RA [18, 48]. Our real-world meta-analysis supports further the reduction in all-cause mortality, suggesting better cardiovascular protection when these two drug classes with different mechanisms of action are used together.

Although the exact mechanisms by which SGLT2is and GLP-1RAs reduce CV and all-cause mortality are not yet fully understood, endothelial dysfunction is a common pathogenetic feature underlying HF, especially with preserved ejection fraction, T2DM, and frailty, and empagliflozin has recently been demonstrated to act via regulation of microRNAs and reduction of mitochondrial oxidative stress [49, 50]. The cardiovascular benefits of empagliflozin were evident through the significant improvement in the 5-m gait speed test within 3 months of treatment in older and frail individuals with T2DM and hypertension [50]. The combination of empagliflozin and liraglutide reduced central systolic blood pressure, perfused boundary region, and arterial stiffness in patients with T2DM to a greater extent than insulin in addition to similar glycemic effects, suggesting that the combination should be preferred over traditional insulin plus metformin in patients with T2DM and high cardiovascular risk [51]. Vascular remodeling is a pathological process in cardiovascular diseases, and GLP-1RAs, including semaglutide, have been shown to reduce vessel remodeling through their anti-inflammatory and anti-proliferative effects independent of their interaction with GLP-1R [52].

The present analysis indicates that simultaneous treatment with the combination of SGLT2is + GLP-1RAs is effective in significantly reducing baseline HbA1c and FPG levels in patients with T2DM at follow-up. Accumulated data underscore the advantages of combining SGLT2is and GLP-1RAs for cardiovascular, renal, and metabolic health in T2DM patients at a low risk of developing hypoglycemia [53]. Individuals newly diagnosed with T2DM frequently present with multiple comorbid conditions that increase their susceptibility to CVD, such as hypertension, dyslipidemia, and obesity [54]. The severity of these comorbidities is especially high in patients after 1 year of being diagnosed with T2DM and presenting with HbA1c levels of > 6.5% [55]. Hence, it is crucial to attain early and sustained glycemic control to prevent diabetes-related complications.

GLP1-RAs promote pancreatic insulin secretion to regulate the levels of glucose in the blood. Apart from their role in insulin secretion, GLP-1RAs delay gastric emptying, stimulate the appetite centers in the brain to induce early satiety, and consequently reduce food consumption [13, 26, 56]. SGLT2is also contribute to weight loss as a result of caloric loss through glycosuria; however, the weight loss may be less than expected due to increased food intake, especially if adequately intensive lifestyle changes are not implemented [57]. A retrospective search of the electronic prescriptions of patients with T2DM (N = 446,798) for SGLT2i + GLP-1RA treatment in Spain (2018) showed that the combination resulted in faster weight loss and greater HbA1c reduction when administered simultaneously than when the drugs were administered sequentially [26]. This real-world data analysis also shows a significant decrease in BMI with the combination therapy. Notably, there was a higher reduction in BMI when an SGLT2i was added to the GLP-1RA therapy rather than vice versa.

A significant decrease in SBP was seen at 6 months follow-up compared to baseline among patients who received simultaneous SGLT2i + GLP-1RA therapy. While no significant difference in the SBP between patients who received the simultaneous versus sequential combination therapy was apparent, the reduction in SBP was greater in patients who first received SGLT2is and then received GLP-1RAs. This is consistent with a previous review that focused on data from placebo-controlled trials regarding the antihypertensive effects of GLP-1RAs, SGLT2is, and DPP-4 inhibitors; this review reported that SGLT2is are more potent than the other two drug classes in reducing SBP and diastolic blood pressure among patients with T2DM [58]. The decrease in SBP can be attributed to the natriuresis induced by SGLT2is [59]. GLP-1RAs exhibit a nephroprotective effect by directly interacting with renal cells; they prevent glomerular hyperfiltration by promoting diuresis and natriuresis [60].

In the present analysis, the difference between the baseline and the follow-up eGFR was statistically non-significant in patients with T2DM who received the combination; however, the duration of follow-up in these real-world studies was not long enough to observe any improvement in kidney function that was demonstrated in larger SGLT2i trials, e.g., the DECLARE TIMI58 trial (median follow-up: 4.2 years [39]) and CANVAS trial (mean follow-up: 3.6 years [38]). More recently, the FLOW study, which focused on evaluating the nephroprotective and cardioprotective efficacy of semaglutide, a GLP-1RA, in patients with T2DM and CKD (N = 3534), was stopped prematurely as it met certain pre-specified efficacy criteria, with a very high likelihood of study success on reducing the progression of renal disease [61]. Microalbuminuria is an independent risk factor for progressive CKD and cardiovascular events, especially in patients with T2DM [62]. Post hoc analyses of the double-blind, placebo-controlled Semaglutide Treatment Effect in People with obesity (STEP) 2 trial, which involved overweight/obese patients with T2DM (N = 1210), showed that those on semaglutide had an improved UACR status. Additionally, treatment with semaglutide led to a reduction in the proportion of patients with microalbuminuria (UACR 30–300 mg/g) compared to treatment with placebo at the end of week 68 (11.5% vs. 22.4%) [63]. SGLT2is are recommended for the treatment of patients with T2DM and CKD (eGFR ≥ 30 mL/min/1.73 m^2^ and UACR > 30 mg/g), given their nephroprotective effects via the renin–angiotensin–aldosterone system [64]. However, for those with impaired renal function (eGFR < 30 mL/min/1.73 m^2^), the lowering of HbA1c levels by SGLT2is is negligible [65]. This again supports the rationale for combining SGLT2is with GLP-1RAs, which exert their glucose-lowering effect independent of kidney function. GLP-1RAs are reported to inhibit the development and/or progression of kidney disease in patients with T2DM while reducing the risk of kidney damage [66].

Estimates for CV mortality could not be derived in this meta-analysis, given the lack of real-world studies reporting CV mortality outcomes and meeting the study eligibility criteria. The Journal of the American College of Cardiology 2020 expert consensus report on the combined use of these drugs suggested the need for more evidence-based studies supporting the use of this combination for its CV benefits but concluded that the concurrent use of both SGLT2is and GLP-1RAs is permissible when indicated, considering the patient benefits established in numerous trials [67]. Guidelines and recommendations from various medical bodies, including the American Diabetes Association (ADA), European Society of Cardiology, and Diabetes Canada, have advocated for adding an SGLT2i following the use of a GLP-1RA, or vice versa, for T2DM patients with a high risk of developing ASCVD and those with CKD [68–70].

Despite the substantial amount of literature proposing the advantages of using SGLT2is + GLP-1RAs, a retrospective cross-sectional two-center study conducted in Riyadh, Saudi Arabia (January–December 2020), showed that physicians were under-prescribing these drugs; the study showed that endocrinologists most frequently prescribed SGLT2is or GLP-1RAs (60.6%), followed by internal medicine physicians (11.4%), cardiologists (9.8%), and nephrologists (2.0%) [71]. While RCT meta-analyses and findings from the present real-world analysis demonstrate the added benefit of combination therapy among patients with T2DM [13, 14, 16, 51, 72–74], very little has truly been translated into practice. Despite recommendations to include the combination in patients with T2DM, cardiologists view diabetes care independently of cardiovascular care, and consequently, are reluctant to prescribe SGLT2is + GLP-1RAs for their cardiovascular benefits in the management of patients with T2DM [75, 76]. Recognizing this reluctance, it is important to analyze the barriers associated with integrating these treatments into routine clinical practice to enhance cardiovascular outcomes [71]. Combination therapy with SGLT2is + GLP-1RAs can address multiple components of the ominous octet (insulin and glucagon secretion, hepatic glucose production, gastrointestinal incretin defect, appetite and weight loss, and muscle and hepatic insulin sensitivity), improve cardiovascular risk, and prevent diabetic nephropathy and should therefore be considered as an option during the early treatment stages of patients with T2DM. Combination therapy should perhaps be considered for first-line treatment in patients with T2DM who are at high risk of cardiovascular and renal disease as it is not associated with any notable safety issues or adverse event outcomes [77]. Treatment goals for patients with T2DM should be focused on the timely control of HbA1c levels along with the prevention of microvascular and macrovascular complications [78].

This systematic review has some limitations. First, publication bias assessment was not possible as relatively few studies were identified for each of the outcomes presented in this SLR. Second, there was considerable heterogeneity in patient demographics (e.g., age), duration of diabetes, medical history (e.g., baseline HbA1c), treatment history, and concomitant medications for T2DM (e.g., insulin, metformin, or other glucose-lowering agents) among the included studies. Most of the studies did not report the duration for which the patients received treatment with SGLT2i or GLP-1RA until add-on was initiated, which would have provided insights into the extent of heterogeneity in the timing of therapy titration. Accordingly, for all the outcomes, the certainty of evidence was deemed low-to–very low in the GRADE analysis. Third, unadjusted mortality values have been presented in the current meta-analysis. Given that both SGLT2is and GLP-1RAs strongly influence BMI and microalbuminuria, it will be interesting to note how mortality varies as a function of these parameters. Such adjustment analyses could not be conducted due to lack of number of studies reporting mortality outcomes. Fourth, a meta-analysis for cardiovascular mortality could not be performed due to the lack of sufficient data. This calls for more and larger-sized real-world studies to strengthen the evidence that supports the early use of these combinations to improve cardiovascular outcomes and glycemic control in patients with T2DM.

## Conclusion

This SLR and meta-analysis of real-world studies suggests that the combination of SGLT2is + GLP-1RAs is associated with significantly lower all-cause mortality than individual therapies, with an improvement in cardiovascular, renal, and glycemic measurements. Providing evidence that supports the advantages of introducing the combination early can significantly strengthen the foundation for making confident clinical decisions. Moreover, the simultaneous use of these drugs could prove more beneficial than sequential combination therapy in patients with T2DM, and if similar results are reported with the use of oral GLP-1RAs, it may be easier to initiate the combination earlier in the disease course.

### Supplementary Information

**Additional file 1: Table S1.** Search strategy for the systematic literature review. **Table S2.** List of studies excluded at the full-text screening stage with reasons for exclusion. **Table S3.** List of studies excluded from the meta-analysis and reasons for exclusion.

**Additional file 2. **Assessment of study quality and data extraction.

## Data Availability

Data sharing is not applicable to this article as no datasets were generated or analyzed.

## Abbreviations

ADA: American Diabetes Association
BMI: Body Mass Index
CKD: Chronic kidney disease
eGFR: Estimated glomerular filtration rate
FPG: Fasting plasma glucose
GLP-1RAs: Glucagon-like peptide-1 receptor agonists
GRADE: Grading of Recommendations, Assessment, Development and Evaluation
JBI: Joanna Briggs Institute
LDL-C: Lipoprotein cholesterol
MACE: Major Adverse Cardiovascular Events
RCTs: Randomized controlled trials
SGLT2is: Sodium–glucose transport protein 2 inhibitors
SBP: Systolic blood pressure
T2DM: Type 2 diabetes mellitus
MD: Mean difference
OR: Odds ratio
