# Correction to: Effectiveness and safety of the combination of sodium–glucose transport protein 2 inhibitors and glucagon-like peptide-1 receptor agonists in patients with type 2 diabetes mellitus: a systematic review and meta-analysis of observational studies

**DOI:** 10.1186/s12933-024-02283-2

**Published:** 2024-06-01

**Authors:** Aftab Ahmad, Hani Sabbour

**Affiliations:** 1 Department of Endocrinology, Imperial College London Diabetes Centre, Abu Dhabi, United Arab Emirates; 2 Department of Endocrinology, Khalifa Medical University, Abu Dhabi, United Arab Emirates; 3 Department of Cardiology, Mediclinic Hospital, Abu Dhabi, United Arab Emirates; 4 Department of Cardiology, Warren Alpert Medical School of Brown University, Providence, RI USA; 5 Department of Cardiology, Imperial College London Diabetes Centre, Abu Dhabi, United Arab Emirates

**Correction to: Cardiovascular Diabetology (2024) 23:99**

10.1186/s12933-024-02192-4

Following publication of the original article [1], the author noticed the errors in Discussion section, Acknowledgement statement, Author contributions and Funding statement. These corrections are given below:

In Discussion section under second paragraph, the sentence should read, “The SOUL trial (Semaglutide Cardivascular Outcomes trial) will provide further evidence regarding the CV effects of oral semaglutide in individuals with type 2 diabetes and established ASCVD and/or CKD [46]” instead of “The SOUL study demonstrated the non-inferiority of semaglutide to placebo in terms of CV mortality outcomes [46]”.

The updated Acknowledgements statement should read, “We would like to thank BioQuest Solutions Pvt. Ltd for providing medical writing support and editorial assistance, and Novo Nordisk, UAE for the unrestricted funding” Instead of “we would like to thank BioQuest Solutions Pvt. Ltd. for providing medical writing support and editorial assistance”.

In Author contributions, the paragraph should read, “All authors have contributed equally and significantly to the conceptualization and design of review, data retrieval analysis and interpretation. Both the authors drafted the article and revised it critically for intellectual content. All authors have read and reviewed the final draft of this manuscript, take responsibility for the integrity and accuracy of this manuscript, and have given their approval for this version to be published” instead of “All authors have contributed equally and significantly to the conceptualization and investigation of the study. All authors have read and reviewed the final draft of this manuscript, take responsibility for the integrity and accuracy of this manuscript, and have given their approval for this version to be published”.

The Funding statement should read, “The convening of the expert’s meetings, medical writing, and editorial assistance were facilitated by an unrestricted grant from Novo Nordisk, UAE, to BioQuest Solutions Pvt. Ltd on behalf of the experts. None of the authors received any direct compensation for their contribution to this work. Novo Nordisk, UAE did not participate in any of the meetings or the drafting of the manuscript”.

## References

[1] Ahmad A, Sabbour H (2024). Effectiveness and safety of the combination of sodium–glucose transport protein 2 inhibitors and glucagon-like peptide-1 receptor agonists in patients with type 2 diabetes mellitus: a systematic review and meta-analysis of observational studies. Cardiovasc Diabetol.

