# Supplementary material for: Effectiveness and safety of the combination of sodium–glucose transport protein 2 inhibitors and glucagon-like peptide-1 receptor agonists in patients with type 2 diabetes mellitus: a systematic review and meta-analysis of observational studies
Source: Cardiovasc Diabetol. 2024 Mar 18;23:99. doi: 10.1186/s12933-024-02192-4 (PMC10949729; doi:10.1186/s12933-024-02192-4)
Supplement: Supplementary file 1 — Additional file 1: Table S1. Search strategy for the systematic literature review. Table S2. List of studies excluded at the full-text screening stage with reasons for exclusion. Table S3. List of studies excluded from the meta-analysis and reasons for exclusion. [file 12933_2024_2192_MOESM1_ESM.docx]

## Table S1. Search strategy for the systematic literature review

**Logic Grid**

| **Concept term 1** | **Concept term 2** | **Concept term 3** |
| --- | --- | --- |
| "Sodium-Glucose Transporter 2 Inhibitors"[Mesh] | “Glucagon-like peptide receptor agonists” | “Diabetes Mellitus, Type 2” [MeSH] |
| “SGLT2 inhibitors” | “Glucagon-like peptide-1 receptor agonists” | “Type II Diabetes mellitus” |
| “SGLT-2 inhibitors” | “GLP-1 receptor agonists” | “Type 2 diabetes  Mellitus” |
| SGLT2i | “GLP 1 receptor agonists” | “type 2 diabetes” |
|  | “GLP1-RA” |  |
|  | “GLP-1 analogues” |  |
|  | “GLP-1 agonist” |  |
|  | “GLP-1RA” |  |

**MEDLINE**

Search date: 19-05-2023

| **Step** | **Search term** | **Number of hits** |
| --- | --- | --- |
| 1 | "Sodium-Glucose Transporter 2 Inhibitors"[MeSH Terms] OR "SGLT2 inhibitors"[All Fields] OR "SGLT-2 inhibitors"[All Fields] OR "sglt2i"[All Fields] OR "sglt2is"[All Fields] | 8,020 |
| 2 | "Glucagon-like peptide receptor agonists"[All Fields] OR "Glucagon-like peptide-1 receptor agonists"[All Fields] OR "glp 1 receptor agonists"[All Fields] OR "glp 1 receptor agonists"[All Fields] OR "GLP1-RA"[All Fields] OR "GLP-1 analogues"[All Fields] OR "GLP-1 agonists"[All Fields] OR "GLP-1RA"[All Fields] | 5,517 |
| 3 | #1 AND #2 | 1,279 |
| 4 | "diabetes mellitus, type 2"[MeSH Terms] OR "Type II Diabetes mellitus"[All Fields] OR "Type 2 diabetes Mellitus"[All Fields] OR "type 2 diabetes"[All Fields] | 2,27,614 |
| 5 | #3 AND #4 | 1,110 |
| 6 | "systematic review"[All Fields] OR "meta analysis"[All Fields] OR "Randomized controlled trial"[All Fields] OR "RCT"[All Fields] OR "Review"[All Fields] | 4,711,432 |
| 7 | #5 NOT #6 | 421 |

**SCOPUS**

Search date: 19-05-2023

| **Step** | **Search term** | **Number of hits** |
| --- | --- | --- |
| 1 | (TITLE-ABS-KEY ( "sodium-glucose transporter 2 inhibitors" [ ) OR TITLE-ABS-KEY ( "sglt2 inhibitors" ) OR TITLE-ABS-KEY ( "sglt-2 inhibitors" ) OR TITLE-ABS-KEY ( sglt2i ) ) | 8,794 |
| 2 | (TITLE-ABS-KEY ( "glucagon-like peptide receptor agonists" ) OR TITLE-ABS-KEY ( "glucagon-like peptide-1 receptor agonists" ) OR TITLE-ABS-KEY ( "glp-1 receptor agonists" ) OR TITLE-ABS-KEY ( "glp 1 receptor agonists" ) OR TITLE-ABS-KEY ( "glp1-ra" ) OR TITLE-ABS-KEY ( "glp-1 analogues" ) OR TITLE-ABS-KEY ( "glp-1 agonist" ) OR TITLE-ABS-KEY ( "glp-1ra" ) ) | 12,140 |
| 3 | (TITLE-ABS-KEY ( "diabetes mellitus, type 2" ) OR TITLE-ABS-KEY ( "type ii diabetes mellitus" ) OR TITLE-ABS-KEY ( "type 2 diabetes mellitus" ) OR TITLE-ABS-KEY ( "type 2 diabetes" ) ) | 244,005 |
| 4 | #1 AND #2 AND #3 | 1,541 |
| 5 | (TITLE-ABS-KEY ( "systematic review" ) OR TITLE-ABS-KEY ( review ) OR TITLE-ABS-KEY ( "meta-analysis" ) OR TITLE-ABS-KEY ( "randomized controlled trial" ) ) | 6,706,150 |
| 6 | #4 NOT #5 | 655 |

**CINHAL**

Search date: 19-05-2023

| **Step** | **Search term** | **Number of hits** |
| --- | --- | --- |
| 1 | TX "Sodium-Glucose Transporter 2 Inhibitors" OR TX “SGLT2 inhibitors” OR TX SGLT2i OR “SGLT-2 inhibitors” | 1,968 |
| 2 | TX “Glucagon-like peptide receptor agonists” OR TX “Glucagon-like peptide-1 receptor agonists” OR TX “GLP-1 receptor agonists” OR TX “GLP 1 receptor agonists” OR TX “GLP1-RA” OR TX “GLP-1 analogues” OR TX “GLP-1 agonist” OR TX “GLP-1RA” | 1,938 |
| 3 | MH “Diabetes Mellitus, Type 2” OR “Type II Diabetes mellitus” OR “Type 2 diabetes Mellitus” OR “type 2 diabetes” | 69,159 |
| 4 | #1 AND #2 AND #3 | 303 |
| 5 | TX systematic review OR TX meta-analysis OR TX (randomized controlled trials or rtc or randomised control trials) OR TX review | 1,561,012 |
| 6 | #4 NOT #5 | 90 |

**Google Scholar**

Search date: 19-05-2023

First 10 pages

| **Step** | **Search term** | **Number of hits** |
| --- | --- | --- |
| 1 | Sodium-Glucose Transporter 2 Inhibitors, Glucagon-like peptide receptor agonists, combination therapy, type 2 diabetes mellitus | 100 |

**PROQuest Thesis and Dissertations**

Search date: 19-05-2023

| **Step** | **Search term** | **Number of hits** |
| --- | --- | --- |
| 1 | abstract(Sodium-Glucose Transporter 2 Inhibitors) OR abstract(SGLT2 inhibitors) OR abstract(SGLT-2 inhibitors) OR abstract(SGLT2i) | 1,549 |
| 2 | abstract(Glucagon-like peptide receptor agonists) OR abstract(Glucagon-like peptide-1 receptor agonists) OR abstract(GLP-1 receptor agonists) OR abstract(GLP 1 receptor agonists) OR abstract(GLP1-RA) OR abstract(GLP-1 analogues) OR abstract(GLP-1 agonist) OR abstract(GLP-1RA) | 1,914 |
| 3 | (Diabetes Mellitus, Type 2) OR (Type II Diabetes mellitus) OR (Type 2 diabetes Mellitus) OR (type 2 diabetes) | 3,83,427 |
| 4 | #1 AND #2 AND #3 | 294 |
| 5 | abstract(systematic review OR meta-analysis OR randomized controlled trials OR review OR RCT) | 458,310 |
| 6 | #4 NOT #5 | 179 |

## Table S2. List of studies excluded at the full-text screening stage with reasons for exclusion

| **Sl. No.** | **Author name and year** | **Title** | **Reason for exclusion** |
| --- | --- | --- | --- |
| 1 | Devineni et al. 2021 | Newer Treatments and Glycemic Control in US Adults With Diabetes Across Risk Groups, Sex, and Ethnicity: The NIH Precision Medicine Initiative (All Of Us Study) | SGLT2i+GLP-1RA group not present |
| 2 | Lopez et al. 2022 | Benefits of Adding Glucagon-Like Peptide 1 Receptor Agonists to Sodium-Glucose Co-Transporter 2 Inhibitors in Diabetic Patients With Atherosclerotic Disease and Heart Failure | Outcomes not matching with protocol |
| 3 | Bhattacharyya et al. 2020 | Clinical effectiveness of combination therapy with dulaglutide, SGLT2 inhibitor and metformin with or without insulin in Indian adults with type 2 diabetes: a real-world retrospective study | Duplicate |
| 4 | Saroka et al. 2015 | SGLT-2 Inhibitor Therapy Added to GLP-1 Agonist Therapy in the Management of T2DM | Duplicate |
| 5 | Gonsalves et al. 2017 | Glucagon‐like peptide‐1 receptor agonists and sodium‐glucose co‐transporter‐2 inhibitors: Sequential or simultaneous start? - Goncalves - 2017 - Diabetes, Obesity and Metabolism - Wiley Online Library | Duplicate |
| 6 | Kim et al. 2021 | Clinical Efficacy of Sodium-Glucose Cotransporter 2 Inhibitor and Glucagon-Like Peptide-1 Receptor Agonist Combination Therapy in Type 2 Diabetes Mellitus: Real-World Study | Duplicate |
| 7 | Schapiro et al. 2023 | Real-World Patterns of Basal Insulin Use with Other Diabetes Medications Among People with Type 2 Diabetes Between 2014 and 2020 | Basal insulin-GLP-1RA combination therapy is studied; not SGLT2i+GLP-1RA combination |
| 8 | DeRemer et al. 2021 | Comparing cardiovascular benefits between GLP-1 receptor agonists and SGLT2 inhibitors as an add-on to metformin among patients with type 2 diabetes: A retrospective cohort study | Combination therapy not studied; comparison between two drugs |
| 9 | Curtis et al. 2016 | Addition of SGLT2 inhibitor to GLP-1 agonist therapy in people with type 2 diabetes and suboptimal glycaemic control | Case note review without results table |
| 10 | Frías et al. 2016 | Exenatide once weekly plus dapagliflozin once daily versus exenatide or dapagliflozin alone in patients with type 2 diabetes inadequately controlled with metformin monotherapy (DURATION-8): a 28 week, multicentre, double-blind, phase 3, randomised controlled trial | Clinical trial |
| 11 | Ludvik et al. 2018 | Dulaglutide as add-on therapy to SGLT2 inhibitors in patients with inadequately controlled type 2 diabetes (AWARD-10): a 24-week, randomised, double-blind, placebo-controlled trial | Clinical trial |
| 12 | Singh et al. 2022 | Metabolic and cardiovascular benefits with combination therapy of SGLT-2 inhibitors and GLP-1 receptor agonists in type 2 diabetes | Review article |
| 13 | Meade et al. 2019 | The Effect of Glucagon-Like Peptide-1 Receptor Agonists and Sodium-Glucose Cotransporter-2 Inhibitors in Patients Prescribed Regular U-500 Insulin. | No data on exclusive use of SGLT2i+GLP-1RA combination |
| 14 | Wright et al. 2022 | Primary Prevention of Cardiovascular and Heart Failure Events With SGLT2 Inhibitors, GLP-1 Receptor Agonists, and Their Combination in Type 2 Diabetes \| Diabetes Care \| American Diabetes Association | Exposure to the combination regimen is studied; no outcomes reported |
| 15 | Riley et al. 2023 | All‐cause mortality and cardiovascular outcomes with sodium‐glucose Co‐transporter 2 inhibitors, glucagon‐like peptide‐1 receptor agonists and with combination therapy in people with type 2 diabetes | Duplicate |
| 16 | Jones et al. 2016 | Evaluation of a combination of SGLT2 inhibitor and GLP-1 receptor agonist treatment in type 2 diabetes | Review article |
| 17 | Edwards et al. 2023 | Clinical and Safety Outcomes With GLP-1 Receptor Agonists and SGLT2 Inhibitors in Type 1 Diabetes: A Real-World Study \| The Journal of Clinical Endocrinology & Metabolism \| Oxford Academic | Studied participants with type 1 diabetes |
| 18 | Hayden et al. 2016 | Evaluation of a combination of SGLT2 inhibitor and GLP-1 receptor agonist treatment in type 2 diabetes - DiabetesontheNet | Review article |
| 19 | Edwards et al. 2023 | Clinical and Safety Outcomes With GLP-1 Receptor Agonists and SGLT2 Inhibitors in Type 1 Diabetes: A Real-World Study | Duplicate |
| 20 | Lambadiari et al. 2021 | Effects of a 12-month treatment with glucagon-like peptide-1 receptor agonists, sodium-glucose cotransporter-2 inhibitors, and their combination on oxidant and antioxidant biomarkers in patients with type 2 diabetes | RCT |
| 21 | Pratley et al. 2020 | 927-P: Effect of Oral Semaglutide with or without Background SGLT2i in Patients with T2D: Subgroup Analysis of PIONEER 4 | Conference abstract |
| 22 | Ikonomidis et al. 2021 | Effects of glucagon like peptide-1 receptor agonists and their combination with sodium-glucose cotransporter-2 inhibitors on myocardial deformation and work index in type 2 diabetes: 1 year follow up | RCT |
| 23 | Sammour et al. 2020 | Effects of GLP-1 Receptor Agonists and SGLT-2 Inhibitors in Heart Transplant Patients with Type 2 Diabetes: a Case Series. | Case series |
| 24 | Jayasinghe et al. 2016 | Combining SGLT2 inhibitor and GLP-1 agonist: Exaggerated weight loss in a morbidly obese patient with type 2 diabetes | Case report |
| 25 | PerssonF et al. 2018 | Different patterns of second-line treatment in type 2 diabetes after metformin monotherapy in Denmark, Finland, Norway and Sweden (D360 Nordic): A multinational observational study. | Prescription based study, comparison between countries |
| 26 | Ghosal et al. 2018 | Liraglutide and Dulaglutide therapy in addition to SGLT-2 inhibitor and metformin treatment in Indian type 2 diabetics: a real world retrospective observational study. | SGLT2i+GLP-1RA group is not present |
| 27 | Warden et al. 2021 | Real-world utilization of pharmacotherapy with new evidence-based cardiovascular indications in an academic preventive cardiology practice. | Based on prescription pattern |
| 28 | Zerovnik et al. 2021 | Cardiovascular morbidity and mortality in patients with type 2 diabetes using novel antidiabetic medicines as add-on therapy: An observational real-world study | SGLT2i+GLP-1RA group is not present |
| 29 | Wang et al. 2023 | Efficacy and safety of glucagon-like peptide-1 receptor agonist combined with sodium-glucose co-transporter-2 inhibitor in the treatment of type 2 diabetes mellitus patients with obesity: a retrospective analysis study | RCT |
| 30 | Akuta et al. 2022 | Favorable impact of long-term SGLT2 inhibitor for NAFLD complicated by diabetes mellitus: A 5-year follow-up study | Case series |
| 31 | Zhao et al. 2022 | Hypoglycemia Risk With SGLT2 Inhibitors or Glucagon-Like Peptide 1 Receptor Agonists Versus Sulfonylureas Among Medicare Insured Adults With CKD in the United States | SGLT2i+GLP-1RA combination is not a study group, |
| 32 | Ohki et al. 2016 | Effectiveness of Ipragliflozin, a Sodium-Glucose Co-transporter 2 Inhibitor, as a Second-line Treatment for Non-Alcoholic Fatty Liver Disease Patients with Type 2 Diabetes Mellitus Who Do Not Respond to Incretin-Based Therapies Including Glucagon-like Peptide-1 Analogs and Dipeptidyl Peptidase-4 Inhibitors | SGLT2i+GLP-1RA alone data not available |
| 33 | Sano et al. 2020 | Efficacy of sodium-glucose cotransporter 2 inhibitor with glucagon-like peptide-1 receptor agonist for the glycemic control of a patient with Prader-Willi syndrome: a case report | Case report |
| 34 | Cessario et al. 2021 | Empagliflozin, alone or in combination with liraglutide, limits cell death in vitro: role of oxidative stress and nitric oxide | Laboratory study |
| 35 | Ciemins et al. 2021 | Cardiovascular disease in patients with type 2 diabetes a qualitative analysis of knowledge, attitudes, and beliefs of health care professionals | Qualitative study |
| 36 | Weng et al. 2019 | The prevalence of cardiovascular disease and antidiabetes treatment characteristics among a large type 2 diabetes population in the United States. | SGLT2i+GLP-1RA combination is not a study group, |
| 37 | Zoupas et al. 2021 | 88-LB: Kidney and Metabolic Benefits by Adding GLP-1 Agonists and/or SGLT2 Inhibitors on Metformin in Obese Type 2 Diabetes (T2DM) Patients: 24-Month Real-World Data from Both Urban and Rural Clinics | Conference abstract |
| 38 | Saeed et al. 2021 | Ventricular-arterial coupling as a potential therapeutic target in diabetes. | Short communication without results |
| 39 | Colosimo et al. 2021 | Effects of antidiabetic agents on steatosis and fibrosis biomarkers in type 2 diabetes: A real-world data analysis | SGLT2i+GLP-1RA combination is not a study group, |
| 40 | Kobayashi et al. 2021 | The Comparison of the Kidney Effects of Dipeptidyl Peptidase 4 Inhibitors and Glucagon-Like Peptide 1 Agonist-Administered Concomitant with Sodium-Glucose Cotransporter 2 Inhibitors in Japanese Patients with Type 2 Diabetes Mellitus and Chronic Kidney Disease | Outcomes not matching with protocol |
| 41 | Østergaard et al. 2023 | Cardiovascular risk and lifetime benefit from preventive treatment in type 2 diabetes: A post hoc analysis of the CAPTURE study | SGLT2i+GLP-1RA combination is not a study group, |
| 42 | Chaudhry et al. 2021 | Acute Pancreatitis-Induced Euglycemic Diabetic Ketoacidosis. | Case report |
| 43 | Clegg et al. 2019 | Effects of exenatide and open-label SGLT2 inhibitor treatment, given in parallel or sequentially, on mortality and cardiovascular and renal outcomes in type 2 diabetes: insights from the EXSCEL trial. | RCT |
| 44 | de Carvalho et al. 2023 | Underprescription of SGLT2i and GLP-1 RA: CAREPRO-T2D (Cardiorenal Protection in Type 2 Diabetes) Cross-Sectional Study. | No exclusive SGLT2i+GLP-1RA group |
| 45 | Kahkoska et al. 2021 | Association between glucagon-like peptide 1 receptor agonist and sodium–glucose cotransporter 2 inhibitor use and COVID-19 outcomes | No exclusive SGLT2i+GLP-1RA group |
| 46 | Zytnick et al. 2021 | 143-LB: Changes in Perceptions and Confidence of Health Care Professionals across Medical Specialties Regarding Use of SGLT2 and GLP-1 for Cardiorenal Benefit in Type 2 Diabetes | Conference abstract |
| 47 | Maranta et al. 2021 | Cardiologist's approach to the diabetic patient: No further delay for a paradigm shift | Review article |
| 48 | Sammour et al. 2021 | Effects of GLP-1 receptor agonists and SGLT-2 inhibitors in heart transplant patients with type 2 diabetes: Initial report from a cardiometabolic center of excellence | Brief communication |
| 49 | Salmen et al. 2023 | The Safety Profile of Sodium-Glucose Cotransporter-2 Inhibitors and Glucagon-like Peptide 1 Receptor Agonists in the Standard of Care Treatment of Type 2 Diabetes Mellitus | SGLT2i+GLP-1RA group is not present |
| 50 | Gao et al. 2020 | Barriers to prescribing glucose-lowering therapies with cardiometabolic benefits | Prescription rate-based study |
| 51 | Patel et al. 2023 | Appropriate Use of SGLT2s and GLP-1RAs with Insulin to Reduce CVD Risk in Patients with Diabetes. | Review article |
| 52 | Nonomura et al. 2020 | SGLT2 inhibitor and GLP-1 receptor agonist combination therapy substantially improved the renal function in a patient with type 2 diabetes: Implications for additive renoprotective effects of the two drug classes | Case report |
| 53 | Rathmann et al. 2018 | Changes in patient characteristics, glucose lowering treatment, glycemic control and complications in type 2 diabetes in general practices (Disease Analyzer, Germany: 2008-2016) | SGLT2i+GLP-1RA group is not present |
| 54 | Morillas et al. 2021 | Insulin withdrawal in diabetic kidney disease: What are we waiting for? | SGLT2i+GLP-1RA group is not present |
| 55 | Alliabi et al. 2022 | Adherence of physicians to evidence-based management guidelines for treating type 2 diabetes and atherosclerotic cardiovascular disease in Ajman, United Arab Emirates | Prescription pattern is studied |
| 56 | Mc et al. 2020 | 960-P: Characteristics of U.S. Patients with Type 2 Diabetes Prescribed GLP-1RA+SGLT2i in Combination during 2018. | Conference abstract |
| 57 | Ohara et al. 2019 | 545-P: The Difference in the Effects on Microalbuminuria Depending on the Order of GLP-1RA and SGLT2i Therapy in Type 2 Diabetes. | Conference abstract |
| 58 | Nargesi et al. 2021 | Contemporary national patterns of eligibility and use of novel cardioprotective antihyperglycemic agents in type 2 diabetes mellitus | Usage pattern of drug studied, no intervention |
| 59 | Pi et al. 2022 | Clinical Efficacy Evaluation and Long-Term Prognosis of Glucagon-Like Peptide-1 Combined with Sodium Glucose Cotransporter-2 Inhibitor in Diabetes | RCT |
| 60 | Candler et al. 2020 | Improvement in glycaemic parameters using SGLT-2 inhibitor and GLP-1 agonist in combination in an adolescent with diabetes mellitus and Prader-Willi syndrome: a case report | Case report |
| 61 | Onishi et al. 2019 | 2345-PUB: Exploring Patient Factors Contributing to Enhanced Efficacy of Incretin-Related Drugs with Add-On SGLT2 Inhibitor Therapy in Reducing Glycemic Variability (GV) | Conference abstract |
| 62 | Trombara et al. 2023 | Impact of chronic GLP-1 RA and SGLT-2I therapy on in-hospital outcome of diabetic patients with acute myocardial infarction | SGLT2i+GLP-1RA intervention is not specifically studied. |
| 63 | Russo et al. 2021 | The “Early Treatment” Approach Reducing Cardiovascular Risk in Patients with Type 2 Diabetes: A Consensus From an Expert Panel Using the Delphi Technique | Delphi study; no intervention |
| 64 | Colosimo et al. 2023 | Improved glycaemic control in patients with type 2 diabetes has a beneficial impact on NAFLD, independent of change in BMI or glucose lowering agent | Exclusive SGLT2i+GLP-1RA group is not present |
| 65 | Iqbal et al. 2023 | Perspectives in weight control in diabetes – SGLT2 inhibitors and GLP-1–glucagon dual agonism | Review article |
| 66 | Al-Ozairi et al. 2023 | Glucagon-like peptide-1 agonists combined with sodium-glucose cotransporter-2 inhibitors reduce weight in type 1 diabetes | Type 1 diabetes |
| 67 | Le et al. 2022 | Combined therapy with sodium-glucose cotransporter-2 inhibitors (sglt2i) and glucagon-like peptide-1 receptor agonists (glp1-ra)—tolerability and clinical impact: the intermountain healthcare real world experience | Full text not available |
| 68 | Kashima et al. 2021 | 402-P: Liraglutide Might Have Synergistic Renal Protection Effect with SGLT2 Inhibitors in Rapidly Progressive Diabetic Kidney Disease | Conference abstract |
| 69 | Blanco et al. 2023 | Use of SGLT2 Inhibitors Reduces Heart Failure and Hospitalization: A Multicenter, Real-World Evidence Study | Hospitalization and heart failure are studied |
| 70 | Kuhadiya et al. 2021 | Effects of concomitant combination of SGLT-2 inhibitor and GLP-1 receptor agonist on renal outcomes in T2D with eGFR below 30 and macroalbuminuria: A case series | Case report |
| 71 | Feher et al. 2022 | Sodium-Glucose Cotransporter-2 Inhibitor and Glucagon-Like Peptide-1 Receptor Agonist Combination Therapy in Type 2 Diabetes: Protocol for a Kidney End Points Real-world Study (COMBi-KID Study) | Protocol; pre study publication |
| 72 | Kajitani et al. 2020 | Severe visceral obesity, fatty liver and diabetes after orchiectomy for prostate cancer | Case report |
| 73 | Tsvetalina et al. 2020 | Familial Hypercholesterolaemia in a Bulgarian Population of Patients with Dyslipidaemia and Diabetes: An Observational Study | Includes T1DM patients |
| 74 | Farmer et al. 2021 | Prescribing in Type 2 Diabetes Patients With and Without Cardiovascular Disease History: A Descriptive Analysis in the UK CPRD | Prescription pattern is investigated |
| 75 | Spiliotis et al. 2020 | 1113-P: The Use of Empagliflozin and Dulaglutide Improves Metabolic and Glycemia Outcomes in a Real-World Primary Care Setting | Conference abstract |
| 76 | Saroka et al. 2015 | SGLT-2 inhibitor therapy added to GLP-1 agonist therapy in the management of T2DM | Duplicate |
| 77 | Horikawa et al. 2018 | Effectiveness of Sodium-Glucose Cotransporter-2 Inhibitor as an Add-on Drug to GLP-1 Receptor Agonists for Glycemic Control of a Patient with Prader–Willi Syndrome: A Case Report | Case report |

## Table S3. List of studies excluded from the meta-analysis and reasons for exclusion

| **Sl. No.** | **Author name and year** | **Title of study** | **Reason for exclusion** |
| --- | --- | --- | --- |
| 1 | Arévalo et al. 2018 | Lowering Blood Pressure with the Combination of a Sodium‑Glucose  Cotransporter 2 Inhibitor and a Glucagon‑like Peptide‑1 Receptor  Agonist in Type 2 Diabetic Patients: A Clinical Evidence | The article reports only median values and not mean values.  Only 2 patients received the combination therapy simultaneously. |
| 2 | Bhattacharyya et al. 2020 | Clinical effectiveness of combination  therapy with dulaglutide, SGLT2 inhibitor  and metformin with or without insulin  in Indian adults with type 2 diabetes:  a real-world retrospective study | Follow-up duration was 3 months. |
| 3 | Deol et al. 2017 | Combination therapy with GLP-1 analogues and SGLT-2 inhibitors  in the management of diabesity: the real world experience | Follow-up period was between 3 and 6 months (mean 4.5 months). |
| 4 | Gorgojo-Martínez et al. 2017 | Real-world effectiveness and safety of dapagliflozin therapy added to a GLP-1  Receptor agonist in patients with type 2 diabetes | Cohort 1 included patients with  background GLP1-RA therapy for at least 6 months and cohort 2 included patients not taking GLP1-RAs. Only differences in outcomes are provided instead of absolute values at baseline and follow-up. |
| 5 | Saroka et al. 2015 | SGLT2 inhibitor therapy added to GLP-1 agonist therapy in the management of T2DM | Only differences in outcomes are provided instead of absolute values at baseline and follow-up. |
