# Supplementary material for: Effectiveness and safety of the combination of sodium–glucose transport protein 2 inhibitors and glucagon-like peptide-1 receptor agonists in patients with type 2 diabetes mellitus: a systematic review and meta-analysis of observational studies
Source: Cardiovasc Diabetol. 2024 Mar 18;23:99. doi: 10.1186/s12933-024-02192-4 (PMC10949729; doi:10.1186/s12933-024-02192-4)
Supplement: Supplementary file 2 — Additional file 2. Assessment of study quality and data extraction. [file 12933_2024_2192_MOESM2_ESM.docx]

# Supplementary Material

## Assessment of study quality

For each domain, studies were scored as "Yes" (1 point) if the criterion was met, or "No" (0 points) if the criterion was not met or the methodology was unclear. The overall quality score for each study was then categorized as low (0–3), moderate (4–7), or high (8–11) based on the total number of points scored across all domains. The quality of the studies included in this SLR ranged from moderate to high. Regardless of the methodological quality of the studies, data extraction and synthesis were carried out for all the studies.

## Data extraction

The data extraction sheets included the metadata of individual studies and domains such as the number of patients, type of intervention, baseline values, follow-up values, and mean follow-up period for individual outcome measures.
